# Exosomes derived from cancer stem cells of gemcitabine-resistant pancreatic cancer cells enhance drug resistance by delivering miR-210

**DOI:** 10.1007/s13402-019-00476-6

**Published:** 2019-11-12

**Authors:** Zhiyong Yang, Ning Zhao, Jing Cui, Heshui Wu, Jiongxin Xiong, Tao Peng

**Affiliations:** 1 Pancreatic Surgery Center, Department of Hepatobiliary and Pancreatic Surgery, Zhongnan Hospital of Wuhan University, Wuhan, China; 2 Department of Gastrointestinal Surgery, Union Hospital, Tongji Medical College, Huazhong University of Science and Technology, Wuhan, China; 3 Department of Pancreatic Surgery, Union Hospital, Tongji Medical College, Huazhong University of Science and Technology, Wuhan, 430022 China

**Keywords:** Pancreatic cancer, Gemcitabine, Drug resistance, Exosomes, Cancer stem cells, MicroRNA-210

## Abstract

**Purpose:**

Gemcitabine (GEM)-based chemotherapy is the first-line treatment for locally advanced pancreatic cancer. GEM resistance, however, remains a significant clinical challenge. Here, we investigated whether exosomes derived from GEM-resistant pancreatic cancer stem cells (CSCs) mediate cell-cell communication between cells that are sensitive or resistant to GEM and, by doing so, regulate drug resistance.

**Methods:**

GEM-sensitive BxPC-3-derived Bx_S_ and PANC-1 pancreatic cancer cells were cultured with exosomes extracted from CSCs isolated from GEM-resistant BxPC-3-derived Bx_R_ cells (Bx_R_-CSC). The effect of exosomes on drug resistance, cell cycle progression, apoptosis and miRNA expression was evaluated in Bx_S_ and PANC-1 cells. Relevant miRNAs associated with GEM resistance were identified and the role of miR-210 in conferring drug resistance was examined in vitro and in vivo.

**Results:**

Bx_R_-CSC-derived exosomes induced GEM resistance, inhibited GEM-induced cell cycle arrest, antagonized GEM-induced apoptosis, and promoted tube formation and cell migration in Bx_S_ and PANC-1 cells. Elevated miR-210 expression levels were detected in Bx_R_-CSCs and Bx_R_-CSC-derived exosomes compared to those in Bx_S_-CSCs and Bx_S_-CSC-derived exosomes. In addition, increased expression levels of miR-210 were observed in Bx_S_ and PANC-1 cells cultured with Bx_R_-CSC-derived exosomes upon exposure to GEM in a dose-dependent manner. Also, a series of biological changes was observed in Bx_S_ cells after transfection with miR-210 mimics, including activation of the mammalian target of rapamycin (mTOR) signaling pathway, and these changes were similar to those triggered by Bx_R_-CSC-derived exosomes.

**Conclusions:**

Our findings suggest that exosomes derived from GEM-resistant pancreatic cancer stem cells mediate the horizontal transfer of drug-resistant traits to GEM-sensitive pancreatic cancer cells by delivering miR-210.

**Electronic supplementary material:**

The online version of this article (10.1007/s13402-019-00476-6) contains supplementary material, which is available to authorized users.

## Introduction

Pancreatic cancer is a common and aggressive primary malignancy, but so far therapeutic management of patients has resulted in limited success. According to the American Cancer Society, there were approximately 460.000 new cases of pancreatic cancer worldwide, with an estimated 430.000 deaths, in 2018 [1]. Gemcitabine (GEM; 20,20-difluoro-20-deoxycytidine), a nucleoside analog, is a first-line drug approved by the US Food and Drug Administration (FDA) that acts by incorporating into genomic DNA to inhibit DNA synthesis [2, 3]. GEM alone or in combination with other drugs has played a key role in the treatment of locally advanced pancreatic cancer over the past decade. However, the effectiveness of GEM is suboptimal due to the development of resistance after prolonged treatment [4]. Several forms of therapeutic intervention have emerged for the management of GEM-resistant pancreatic cancer. Downregulation of nutrient-deprivation autophagy factor-1 induced by resveratrol has, for example, been found to enhance the sensitivity of pancreatic cancer cells to GEM via NRF2 signaling [5], and similar effects have been observed for metformin [6]. Despite this, the molecular mechanisms underlying progression to GEM resistance in pancreatic cancer still require further exploration.

Tumors and their microenvironments contain multiple types of cells including adult stem cells, stromal cells and cancer stem cells (CSCs), which are known to intercommunicate with each other, thereby modulating tumor progression. CSCs constitute a distinct subpopulation of tumor cells that exhibit multi-lineage differentiation potential and the ability to self-renew. They are associated with cancer initiation, recurrence, and metastasis [7], and in many cases they show an increased resistance to radiation and chemotherapy [8]. Given their ability to accelerate tumor growth, CSCs form a major therapeutic challenge. Although interactions of CSCs with their microenvironment have been shown to be critical for the acquisition of drug resistance [9], the exact underlying cellular and molecular mechanisms remain to be elucidated. Recent studies have provided evidence that exosomes may mediate interactions among different types of cells to enhance cell-cell communication within the tumor microenvironment [10–12]. Hence, exosome signaling may provide new insights into how CSCs may confer drug resistance between drug-resistant and drug-sensitive cells.

Exosomes are small lipid bilayer vesicles with a diameter of 30–120 nm secreted by various cell types and are taken up by neighboring or distant cells to develop mutually supportive positive feedback loops of cellular communication [13, 14]. The main function of exosomes is to participate in cell-cell communication by transferring proteins, DNA, mRNAs, miRNAs and lncRNAs [11, 15, 16]. Exosome-mediated transfer of oncogenic miRNAs from cancer cells may alter the biology of non-cancerous cells, while the transfer of tumor-suppressing miRNAs may inhibit tumor growth [17]. Thus, we hypothesized that CSC-derived exosomes may play a role in chemoresistance in pancreatic cancer by delivering miRNAs.

In the present study, we examined whether CSCs derived from GEM-resistant BxPC-3 cells (Bx_R_) can induce exosome-mediated GEM resistance in GEM-sensitive BxPC-3 cells (Bx_S_). We further investigated whether exosomes derived from Bx_R_-CSCs can mediate horizontal transfer of drug-resistant traits in Bx_S_ and PANC-1 cells by delivering miR-210.

## Materials and methods

### Cells and culture conditions

GEM-sensitive and GEM-resistant BxPC-3 human pancreatic cancer-derived cell lines (Bx_S_ and Bx_R_, respectively) were kindly provided Dr. Yiwei Li (Karmanos Cancer Institute, Wayne State University, Detroit, MI, USA). The human pancreatic cancer cell line PANC-1 was provided by the Stem Cell Bank, Chinese Academy of Sciences. The human pancreatic cancer cells were grown in Dulbecco’s modified Eagle medium (DMEM) supplemented with 5% fetal bovine serum (FBS), 100 units/ml penicillin, and 100 g/ml streptomycin at 37 °C in a humidified atmosphere with 5% CO_2_. Bx_R_ cells were developed from Bx_S_ cells as described previously [18, 19] via exposure to increasing concentrations (up to 200 nM) of GEM. In addition, CD133^+^CD44^+^ CSCs were derived from Bx_S_ and Bx_R_ cells (denoted as Bx_S_-CSCs and Bx_R_-CSCs, respectively) by flow cytometry using antibodies directed against the respective stem cell markers, as described previously [20]. The CSCs were cultured in DMEM supplemented with 10% FBS, 2 mM L-glutamine, penicillin (50 IU/ml), and streptomycin (50 mg/ml) at 37 °C in a humidified atmosphere containing 5% CO_2_. Cells were harvested by trypsinization and washed with phosphate-buffered saline (PBS).

### Identification of CSCs and sphere formation assay

CSCs were cultured in 6-well plates overnight. On the next day, the cells were harvested, centrifuged for 5 min, and resuspended in PBS containing 10% FBS. The resulting cell suspensions (300 μl) were incubated with anti-sex determining region Y-box 2 (SOX-2)-Alexa Fluor®488, anti-octamer-binding transcription factor 4 (OCT-4)-Alexa Fluor®488, and anti-Nanog-Alexa Fluor®488 (all from Thermo Fisher Scientific, Waltham, MA, USA) in the dark at room temperature for 30 min. The proportions of positive cells were quantified using flow cytometry (Beckman, Brea, CA, USA). For the sphere formation assay, CSCs were seeded in 6-well ultralow-cluster plates (Corning Inc., Corning, NY, USA) and cultured for 10 days in serum-free DMEM/F12 (Invitrogen, Carlsbad, CA, USA) supplemented with 2% B27 (Invitrogen), 20 ng/ml epidermal growth factor, 20 ng/ml basic fibroblast growth factor (PeproTech, Offenbach, Germany), 0.4% bovine serum albumin (Sigma), and 5 μg/ml insulin. After formation, the spheres were photographed using a bright-field microscope.

### Isolation and characterization of CSC-derived exosomes

Exosomes were isolated from the supernatants of CSCs using an ExoQuick-TC Kit (System Biosciences, Palo Alto, CA, USA) according to the manufacturer’s instructions. The supernatants were centrifuged at 10,000×g for 30 min to remove cell debris. Next, an ExoQuick-TC Exosome Precipitation Solution was added to the filtered solution at a 1:5 ratio and stored at 4 °C for at least 12 h. Then, the mixture was centrifuged at 1500×g for 30 min and the supernatant was aspirated. The residual solution was centrifuged at 1500×g for 5 min and removed. The extracted exosomes (derived from Bx_S_-CSCs and Bx_R_-CSCs, denoted as Bx_S_-CSCs/Exo and Bx_R_-CSCs/Exo, respectively) were dissolved in PBS and stored at −80 °C. Proteins in the exosomes were extracted using a radioimmunoprecipitation assay lysis buffer (Thermo Fisher Scientific), and expression of the exosome markers CD81, CD63 and GM130 was assessed by Western blotting. The morphology of the exosomes was evaluated by transmission electron microscopy, as described previously [21].

### Pretreatment of Bx_S_ and PANC-1 cells with exosomes

Bx_S_ and PANC-1 cells (~5 × 10^5^) were seeded in 6-well culture plates at 50–60% confluence. Based on the exosomal protein concentration determined by a bicinchoninic acid assay (Sangon Biotech Ltd., Shanghai, China), 2 μg of exosomes were added to each well and cultured for 24 h. To confirm the successful uptake of exosomes, fluorescence labeling of exosomes was performed using a PKH67 labeling kit (MIDI67-1KT, Sigma-Aldrich, St. Louis, MO, USA). Isolated exosomes were diluted and mixed in 1 ml of diluent and incubated with 4 μl of PKH67 solution for 4 min. Next, 2 ml of 0.5% bovine serum albumin in PBS was added to quench the dye, and the exosomes were centrifuged twice at 100,000×g at 4 °C for 70 min each. Bx_S_ and PANC-1 cells were seeded in 6-well plates at 1 × 10^5^ cells/well, and 10 μg of exosomes was added to each well. After overnight incubation at 37 °C in an atmosphere containing 5% CO_2_, the nuclei of the cells were stained with DAPI and the cells were visualized using a fluorescence microscope. Green fluorescence represents PKH67-labeled exosomes.

### Cell viability assay

Cell viability was determined using a 3-(4,5-dimethylthiazol-2-yl)-2,5-diphenyltetrazolium bromide (MTT) assay according to the manufacturer’s recommendations (Sigma-Aldrich). Cells were seeded in a 96-well plate at a density of 5 × 10^3^ cells/well. After treatment, the cells were incubated with 5 mg/ml MTT for 4 h at 37 °C. Thereafter, the culture medium was carefully removed and 200 μl of dimethyl sulfoxide (Sigma-Aldrich) was added to each well at room temperature for 30 min. The absorbance of each well was measured at 570 nm using a microplate reader (Sunrise Microplate Reader, TECAN, Männedorf, Switzerland).

### Cell cycle and apoptosis analyses by flow cytometry

For cell cycle analysis, the harvested cells were washed with PBS and incubated with 10 mg/ml RNase A, 400 mg/ml propidium iodide (PI), and 0.1% Triton-X in PBS at room temperature for 30 min. The stained cells were analyzed by flow cytometry. Cell apoptosis was analyzed using the Annexin V/PI staining method according to the manufacturer’s instructions (Sangon Biotech Ltd.). Cells were seeded in 6-well plates at 50–60% confluence for 24 h. After treatment, the cells were harvested by trypsin, resuspended in PBS at a concentration of 1 × 10^5^ cells/ml, and labeled with 5 μl Annexin V-fluorescein isothiocyanate for 15 min at room temperature. After the addition of 5 μl of PI, the samples were analyzed by flow cytometry (BD Biosciences, Franklin Lakes, NJ, USA). A minimum of 2 × 10^4^ cells was acquired for each sample. Three individual replicates were performed for each experiment.

### Cell invasion and tube formation assays

A Transwell assay was performed to assess cell invasion. Bx_S_ or PANC-1 cells (5 × 10^4^ cells/well) were seeded in the upper chamber insert with 200 μl of serum-free medium and pretreated with CSC-derived exosomes (100 mg/ml), while the lower chamber insert contained complete culture medium with a high concentration of serum to trap invading cells. After treatment with GEM at different concentrations (0, 0.5, 1.5, or 15 μM) for 48 h, cells that penetrated the Matrigel-coated membrane and migrated into the lower chamber were stained with crystal violet (0.1%) and photographed. In each sample, the invasion ability was quantified by counting crystal violet-stained cells. Tube formation assays were performed as previously described [22]. A 24-well plate was coated with 200 μl of Matrigel (BD Biosciences) and incubated at 37 °C for 1 h to form gels. Next, Bx_S_ or PANC-1 cells were seeded onto the Matrigel-coated wells at 2 × 10^4^ cells/well in M199 medium supplemented with low-serum growth supplement. The cells were treated with conditioned media collected from Bx_S_ or PANC-1 cells stimulated with Bx_S_- or Bx_R_-CSC-derived exosomes (100 mg/ml), exposed to different concentrations of GEM (0, 0.5, 1.5, or 15 μM) for 48 h, and evaluated using a bright-field microscope.

### Western blot analysis

Total protein was extracted using a radioimmunoprecipitation assay buffer supplemented with a proteinase inhibitor cocktail (Roche, Basel, Switzerland), after which the protein concentrations were quantified using a bicinchoninic acid assay (Thermo Fisher Scientific). Approximately 30 μg of protein samples were separated by 10% sodium dodecyl sulfate-polyacrylamide gel electrophoresis and transferred to polyvinylidene fluoride membranes (Millipore, CA). Non-specific binding was blocked by incubating the membranes with 5% skim milk for 1 h at room temperature. The membranes were incubated overnight at 4 °C with antibodies directed against SOX-2 (1:1000, Ab97959, Abcam, Cambridge, UK), OCT-4 (1:1000, Ab181557, Abcam), Nanog (1:1000, Ab109250, Abcam), multidrug resistance protein 1 (MDR1, 1:1000, Ab133706, Abcam), Y box binding protein 1 (YB-1, 1:1000, Ab76149, Abcam), breast cancer resistance protein (BCRP, 1:1000, Ab207732, Abcam), CD63 (1:2000, Ab59479, Abcam), CD81 (1:1000, Ab109201, Abcam), GM130 (1:1000, Ab169276, Abcam), cleaved caspase-3 (1:500, Ab13847, Abcam), Bax (1:5000, Ab32503, Abcam), Bcl-2 (1:1000, Ab196495, Abcam), cyclin D1 (1:2000, Ab134175, Abcam), cyclin E (1:2000, Ab71535, Abcam), p27^kip1^ (1:1000, Ab62364, Abcam), mammalian target of rapamycin (mTOR, 1:1000, Ab32028, Abcam), p-mTOR (1:2000, Ab109268, Abcam), ribosomal protein S6 kinase beta-1 (S6K1, 1:5000, Ab32529, Abcam), p-S6K1 (1:1000, Ab2571, Abcam) and GAPDH (1:1000, 2118, CST). After washing, the membranes were incubated with secondary antibodies (anti-mouse IgG, 1:10000, 14,709; anti-rabbit IgG, 1:10000, #14708, all from Cell Signaling Technology, Danvers, MA, USA) for 1 h and visualized using an enhanced chemiluminescence system (Tiangen, Beijing, China).

### Cell transfections

Mimics and inhibitors of miR-210 were designed and synthesized by Sangon Biotech Ltd. Negative control sequences of mimics and inhibitors were designed to be similar and were denoted as miR-NC. Bx_S_ cells in logarithmic growth phase were cultured in a 12-well plate at a density of 2 × 10^4^ cells/well for 24 h. Bx_S_ cells were transfected with 20 nM miR-210 mimics and 25 nM miR-210 inhibitors using Lipofectamine 2000 (Invitrogen) for 48 h according to the manufacturer’s instructions.

### Isolation and detection of miRNAs

Total RNA enriched with miRNAs was isolated from cells or exosomes using a *mir*Vana™ miRNA Isolation Kit (AM1561, Thermo Fisher Scientific). Next, quantitative reverse-transcription polymerase chain reaction (qRT-PCR) was performed following the manufacturer’s instructions to examine the expression of miR-145, miR-210, miR-509, and miR-1243. U6 was used as an internal control. qRT-PCR results were analyzed to obtain Ct values of the amplified products, and the data were analyzed using the 2^-ΔΔCt^ method.

### Tumor formation in a nude mouse model

Nude BALB/c mice (6 weeks old) were purchased from the Guangdong Medical Laboratory Animal Center (Guangzhou, China). All experimental procedures were conducted in accordance with the Chinese legislation regarding experimental animals and were approved by the Ethics Committee of Huazhong Agricultural University (No. HZAUMO-2017-026). Mice were inoculated subcutaneously with Bx_S_ cells (5 × 10^6^). After seven days of tumor growth, the mice were randomized into eight groups (*n* = 8 per group): Bx_S_ (no treatment), Bx_S_ + Bx_R_-CSCs/Exo, Bx_S_ + miR-210 mimics (20 nM), Bx_S_ + miR-210 inhibitors (25 nM), and the corresponding groups with 40 mg/kg GEM treatment. Exosomes, miR-210 mimics, and miR-210 inhibitors were directly administered to the mice via tail vein injections. PBS was used as a vehicle control. GEM was administered by intraperitoneal injection three times a week. The mice were monitored every two days for tumor formation using calipers. The tumor volume was calculated using the following formula: tumor volume (mm^3^) = 0.5 × (*W*)^2^ × (*L*), where *L* represents the length and *W* represents the width of the tumor.

### Statistical analysis

The experimental data are expressed as the mean ± standard deviation (SD) of three independent replicates. Statistical differences between two groups were evaluated by the two-tailed Student’s t test. Those between more than two groups were subjected to analysis of variance (ANOVA) using SPSS 16.0 software, after which the post hoc test of least significant difference was carried out. *P* < 0.05 was considered statistically significant.

## Results

### CSCs derived from Bx_R_ cells exhibit resistance to GEM in vitro

**Fig. 1:**
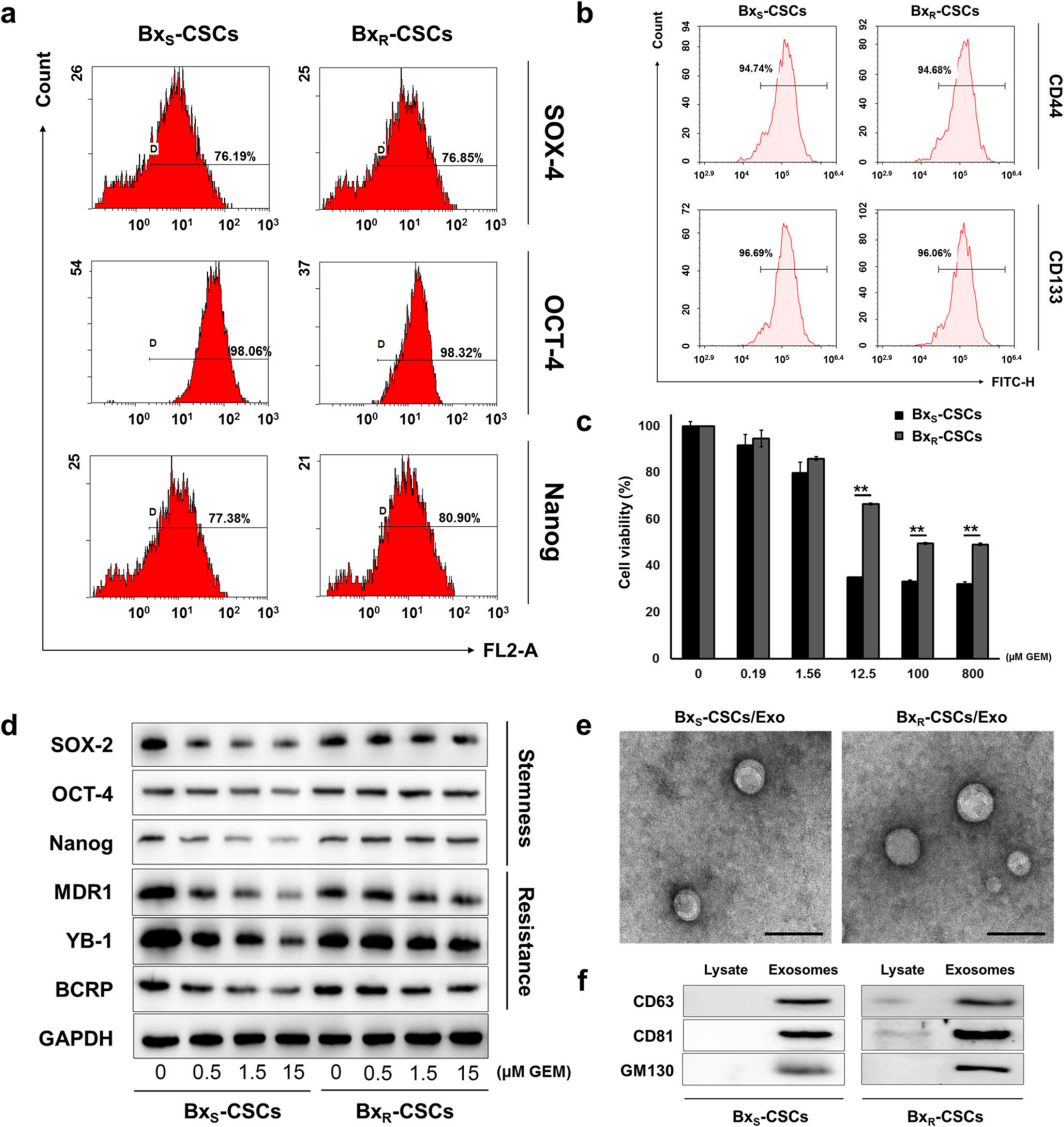
Characterization of CSCs in GEM-resistant BxPC-3 cells (Bx_R_) and isolation of exosomes from CSCs. (**a**) Flow cytometry of CSC self-renewal markers SOX-2, OCT-4, and Nanog in Bx_S_-CSCs and Bx_R_-CSCs. (**b**) Flow cytometric identification of Bx_S_-CSCs and Bx_R_-CSCs using stem cell markers CD44 and CD133. (**c**) MTT viability assay of Bx_S_-CSCs and Bx_R_-CSCs after treatment with GEM at various concentrations (0 to 800 μM) for 48 h. Statistical analysis was carried out using ANOVA. Data are presented as mean ± SD (n = 3), **p < 0.01. (**d**) Western blot analysis of stemness-related proteins SOX-2, OCT-4, and Nanog and drug resistance proteins MDR1, YB-1, and BCRP after treatment of Bx_S_-CSCs and Bx_R_-CSCs with increasing doses of GEM (0 to 15 μM) for 48 h. (**e**) Transmission electron microscopy analysis of the morphology of exosomes derived from Bx_S_-CSCs and Bx_R_-CSCs, scale bar = 200 nm. (**f**) Evaluation of exosome markers CD63, CD81, and GM130 in cell lysates and exosomes derived from Bx_S_-CSCs and Bx_R_-CSCs

To investigate the mechanism of GEM resistance in human pancreatic cancer cells, GEM-resistant pancreatic cancer-derived cell lines have previously been generated, after which CSCs were isolated from GEM-sensitive and GEM-resistant BxPC-3 cells (denoted as Bx_S_ and Bx_R_, respectively). CSCs derived from both Bx_S_ and Bx_R_ cells (Supplementary Fig. S1) showed a positive expression of several CSC self-renewal markers, i.e., SOX-2, OCT-4, and Nanog (Fig. 1a), as well as the stem cell markers CD44 and CD133 (Fig. 1b), confirming that the isolated cell populations were mainly composed of CSCs. To evaluate the involvement of CSCs in the regulation of GEM resistance in pancreatic cancer cells, Bx_S_-CSCs and Bx_R_-CSCs were treated with GEM at 0, 0.19, 1.56, 12.5, 100, or 800 μM for 48 h. A subsequent MTT assay revealed that Bx_R_-CSCs were indeed more resistant to GEM than Bx_S_-CSCs, and that the IC_50_ value of GEM in Bx_R_-CSCs was >50-fold higher than that in Bx_S_-CSCs (Fig. 1c and Supplementary Fig. S2). In addition, the expression of the CSC self-renewal markers SOX-2, OCT-4 and Nanog, and the drug resistance-related proteins MDR1, YB-1 and BCRP decreased gradually as the concentration of GEM increased from 0 to 15 μM in Bx_S_-CSCs, but not in Bx_R_-CSCs (Fig. 1d). Collectively, these data suggest that Bx_R_-CSCs exhibit resistance to GEM, which may be the major cause of the resistance of Bx_R_ cells to GEM.

### Exosomes derived from Bx_R_-CSCs enhance resistance of Bx_S_ cells to GEM

**Fig. 2:**
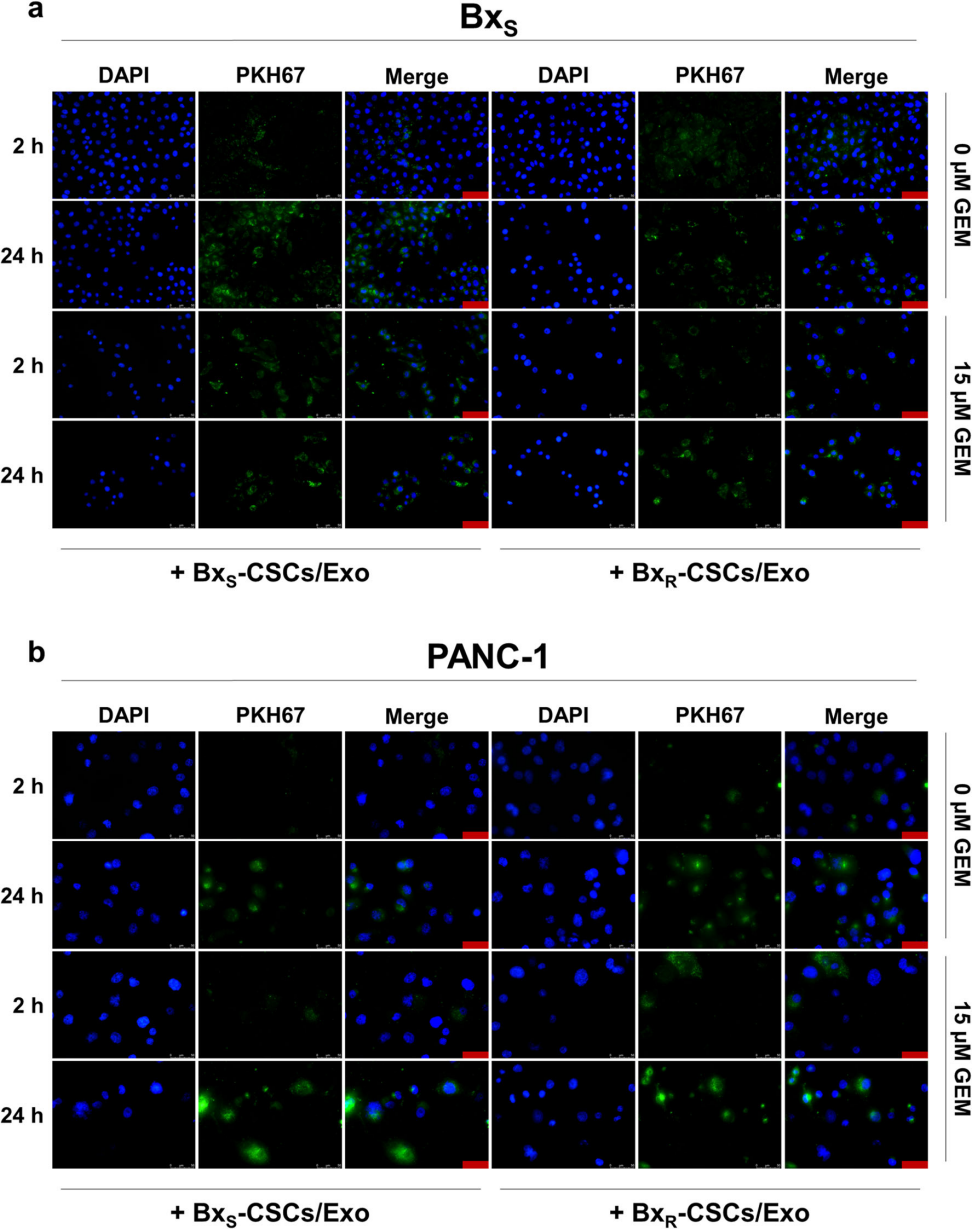
Uptake of exosomes derived from Bx_S_-CSCs and Bx_R_-CSCs by Bx_S_ and PANC-1 cells in the absence or presence of GEM. PKH67 labeling was performed on Bx_S_-CSCs/Exo and Bx_R_-CSCs/Exo. (**a**) Bx_S_ and (**b**) PANC-1 cells were cultured with fluorescently tagged exosomes. Images were acquired 2 h and 24 h after culture. Blue represents DAPI for nuclear staining and green represents PKH67-labeled exosomes. Cells that have endocytosed exosomes emit bright green fluorescence signals. Scale bar = 50 μm

Exosomes ranging from approximately 50–100 nm in diameter were isolated from Bx_S_-CSCs and Bx_R_-CSCs, as demonstrated by transmission microscopy (Fig. 1e). The exosomal protein markers CD63, CD81 and GM130 [23] were strongly expressed in the exosomes, whereas extremely weak expression levels were detected in cell lysates from which the exosomes were isolated (Fig. 1f), further indicating successful exosome extraction. To investigate the effect of CSC-derived exosomes on pancreatic cancer cells, we cultured Bx_S_ or PANC-1 cells with exosomes derived from Bx_R_-CSCs (Bx_R_-CSCs/Exo) or Bx_S_-CSCs (Bx_S_-CSCs/Exo) and treated the cells with GEM at 0, 0.5, 1.5, or 15 μM. First, we confirmed the successful uptake of both types of exosomes by Bx_S_ and PANC-1 cells via PKH67 labeling. Fluorescently labeled exosomes were endocytosed by both Bx_S_ (Fig. 2a) and PANC-1 (Fig. 2b) cells in the absence or presence of GEM (15 μM), as revealed by green fluorescence signals. In addition, we noted accumulation of exosomes over time indicated by the higher fluorescence intensities after 24 h culture than that after 2 h.

**Fig. 3:**
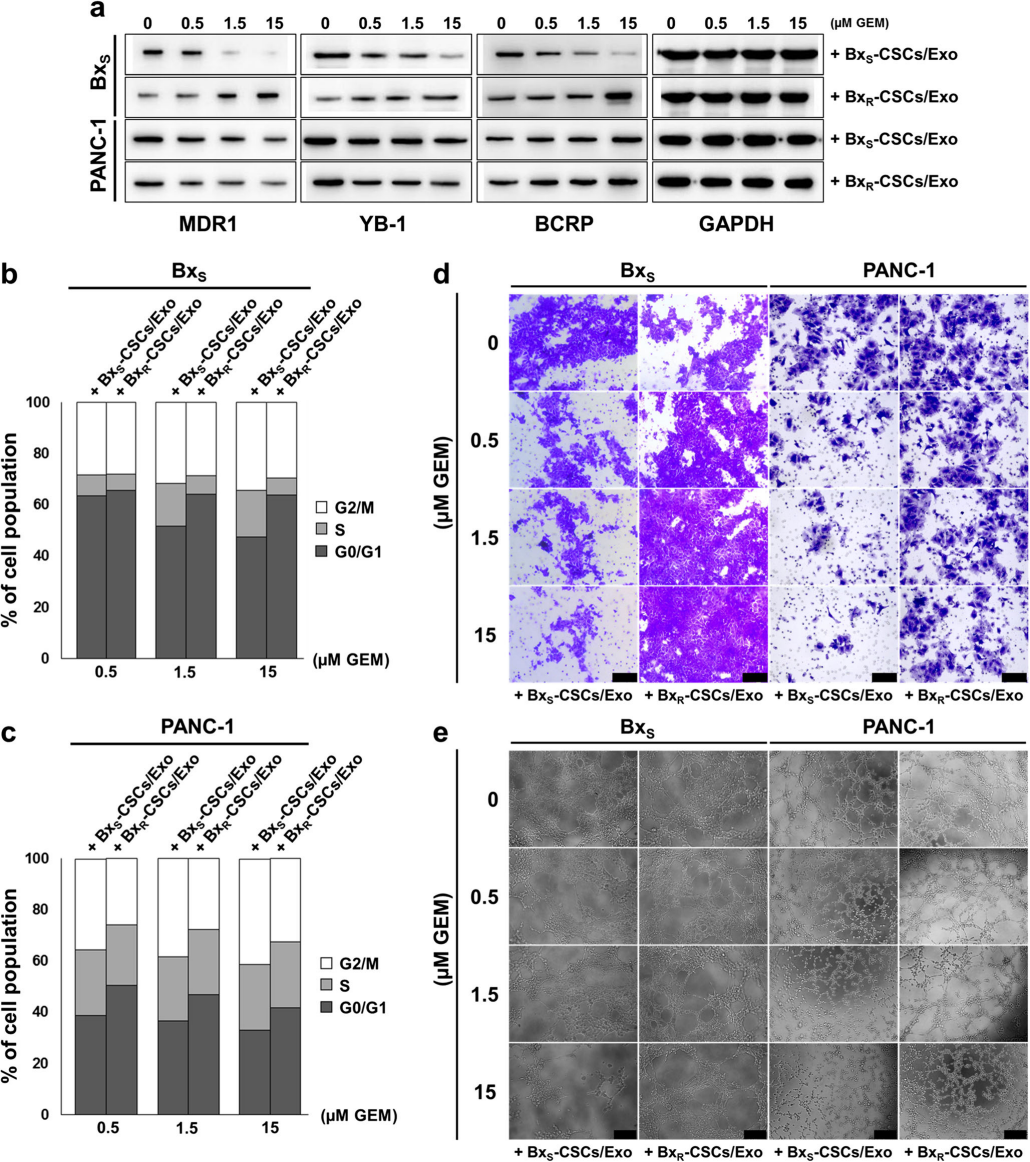
Exosomes derived from Bx_R_-CSCs enhance the resistance of Bx_S_ cells to GEM. (**a**) Western blot analysis of drug resistance markers MDR1, YB-1, and BCRP in Bx_S_ or PANC-1 cells cultured with exosomes from Bx_S_-CSCs or Bx_R_-CSCs and next treated with GEM at various concentrations (0 to 15 μM) for 48 h. Flow cytometric analysis of cell cycle progression in (**b**) Bx_S_ or (**c**) PANC-1 cells cultured with exosomes isolated from Bx_S_-CSCs or Bx_R_-CSCs and next treated with GEM at various concentrations (0 to 15 μM) for 48 h. (**d**) Transwell cell migration assay (scale bar = 50 μm) and (**e**) bright-field microscopy (scale bar = 200 μm) of tube formation in Bx_S_ or PANC-1 cells cultured with exosomes isolated from Bx_S_-CSCs or Bx_R_-CSCs and next treated with GEM at various concentrations (0 to 15 μM) for 48 h

Next, we evaluated the expression of the drug resistance-related proteins MDR1, YB-1 and BCRP in both cell types (Fig. 3a). In Bx_S_ cells cultured with exosomes from Bx_S_-CSCs, each protein was downregulated in a GEM dose-dependent manner, indicating loss of resistance to GEM at gradually increasing concentrations. When Bx_R_-CSCs/Exo were added, however, the resistance-associated proteins were strongly upregulated as the dose of GEM increased, suggesting that exosomes from Bx_R_-CSCs infer drug resistance to Bx_S_ cells. In PANC-1 cells, the same trend was observed when Bx_S_-CSCs/Exo were added, whereas Bx_R_-CSCs/Exo did not prominently affect PANC-1 resistance to GEM.

Subsequently, we closely examined the impact of the two types of exosomes on the survival and proliferation of Bx_S_ and PANC-1 cells. In both cell types, cell cycle arrest at the G0/G1 phase was observed through exposure to GEM after treatment with Bx_R_-CSCs/Exo, but not in cells treated with Bx_S_-CSCs/Exo (Fig. 3b and c, Supplementary Fig. S3 and S4). Furthermore, GEM-induced apoptosis was markedly inhibited by Bx_R_-CSCs/Exo, whereas this inhibition was not observed in the presence of Bx_S_-CSCs/Exo in both cell types (Table 1, Supplementary Fig. S5 and S6). Next, tube formation and migration abilities of Bx_S_ and PANC-1 cells were evaluated in the presence of GEM and CSC-derived exosomes. Bx_R_-CSCs/Exo enhanced the migration and tube formation abilities of Bx_S_ and PANC-1 cells compared to Bx_S_-CSCs/Exo, even at high concentrations of GEM (Fig. 3d and e). Together, these data indicate that Bx_R_-CSCs/Exo can transfer properties of Bx_R_ cells to Bx_S_ and PANC-1 cells by effectively conferring GEM resistance at various drug concentrations.

**Table 1:** Apoptosis of Bx_S_ and PANC-1 cells after treatment with Bx_S_-CSCs/Exo or Bx_R_-CSCs/Exo, with exposure to GEM at various concentrations

		Bx_S_		PANC-1	
μM GEM		+ Bx_S_-CSCs /Exo	+ Bx_R_-CSCs /Exo	+ Bx_S_-CSCs /Exo	+ Bx_R_-CSCs /Exo
0.5	Early (%)	7.25	5.89	3.59	3.99
	Late (%)	5.52	4.11	12.21	6.32
	Total (%)	12.77	10.00	15.80	10.31
1.5	Early (%)	8.89	6.11	3.25	3.98
	Late (%)	20.89	6.15	23.17	7.27
	Total (%)	29.78	12.26	26.42	11.25
15	Early (%)	6.60	6.37	2.71	3.94
	Late (%)	36.24	8.22	34.98	8.04
	Total (%)	42.84	14.59	37.69	11.98

### Exosomes mediate horizontal transfer of miR-210 between Bx_R_ and Bx_S_ cells

**Fig. 4:**
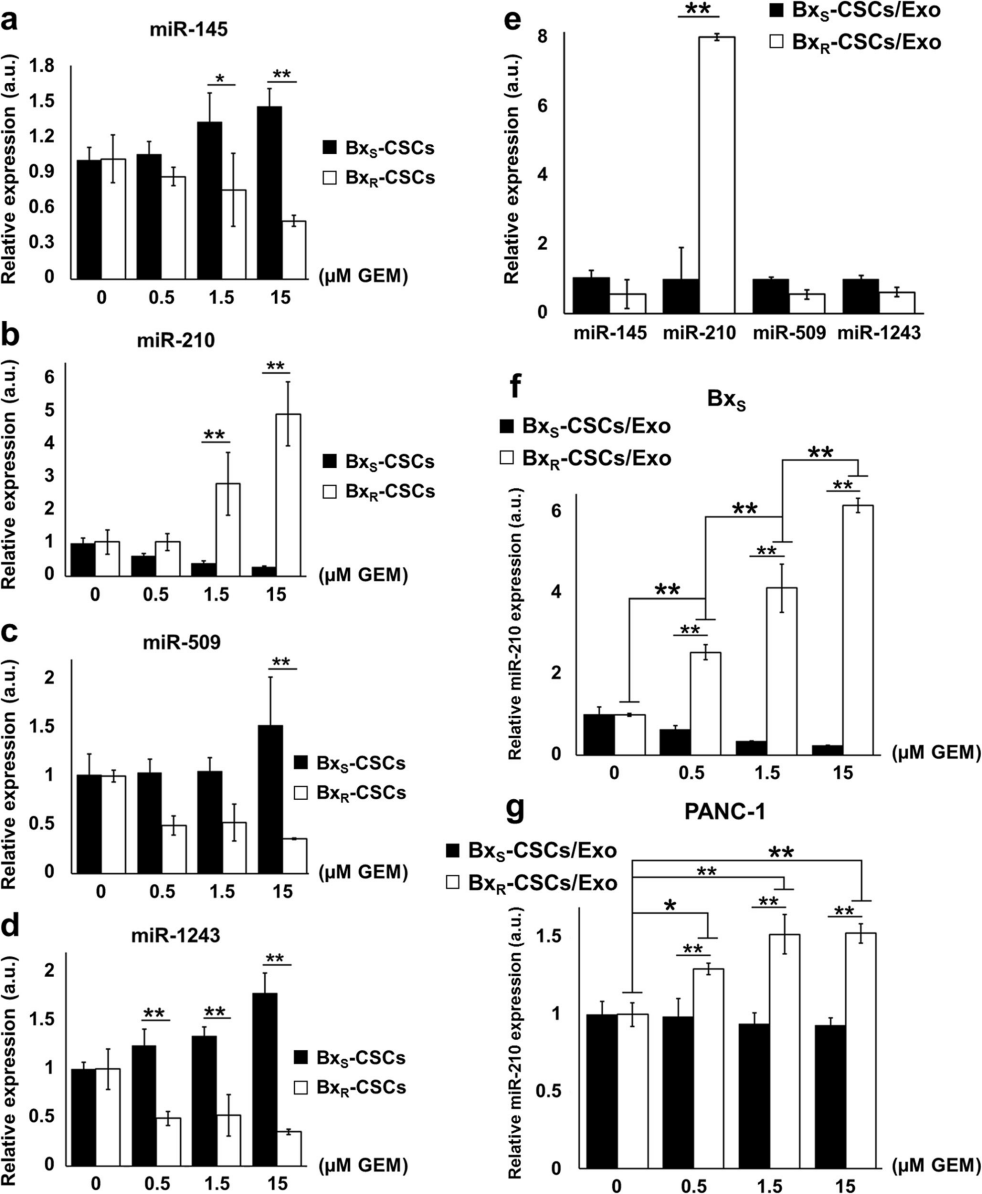
Exosomes mediate horizontal transfer of miR-210 from Bx_R_ to Bx_S_ and PANC-1 cells. Bx_S_-CSCs and Bx_R_-CSCs were exposed to GEM at the indicated concentrations (0 to 15 μM) for 48 h, followed by qRT-PCR to detected expression of (**a**) miR-145, (**b**) miR-210, (**c**) miR-509, and (**d**) miR-1243. (**e**) Expression of miR-145, miR-210, miR-509, and miR-1243 detected by qRT-PCR in exosomes derived from Bx_S_-CSCs and Bx_R_-CSCs (Bx_S_-CSCs/Exo and Bx_R_-CSCs/Exo, respectively). Expression of miR-210 in (**f**) Bx_S_ or (**g**) PANC-1 cells cultured with exosomes from Bx_S_-CSCs or Bx_R_-CSCs and, next, treated with GEM at various concentrations (0 to 15 μM) for 48 h. Statistical analysis was carried out using ANOVA. Data are presented as the mean ± SD (n = 3), ^*^p < 0.05 and ^**^p < 0.01

Exosomes may contain miRNAs that are involved in the regulation of tumor characteristics such as drug resistance [24–26]. To elucidate the molecular mechanisms underlying the role of Bx_R_-CSCs/Exo in conferring drug-resistant traits to Bx_S_ cells, the expression of several drug resistance-related miRNAs reported in previous studies, i.e., miR-145, miR-210, miR-509, and miR-1243, was assessed in CSC-derived exosomes (Fig. 4a–d). The expression of miR-210 increased in a GEM dose-dependent manner in Bx_R_-CSCs compared to that in Bx_S_-CSCs (Fig. 4b). In contrast, no significant differences were noted in the expression of miR-145, miR-509, and miR-1243 (Fig. 4a, c, and d, respectively) when Bx_R_-CSCs were treated with different concentrations of GEM. Moreover, the expression of miR-210, but not of the other miRNAs, was significantly higher in Bx_R_-CSCs/Exo than that in Bx_S_-CSCs/Exo (Fig. 4e). We also found that treatment of Bx_S_ and PANC-1 cells with Bx_R_-CSCs/Exo resulted in an increase in miR-210 expression compared to Bx_S_-CSCs/Exo treatment, and that this increase was dependent on the GEM dose (Fig. 4f and g). These results indicate that exosomes can mediate horizontal transfer of miR-210 between Bx_R_ and Bx_S_/PANC-1 cells.

### miR-210 mediates the resistance of Bx_S_ cells to GEM

**Fig. 5:**
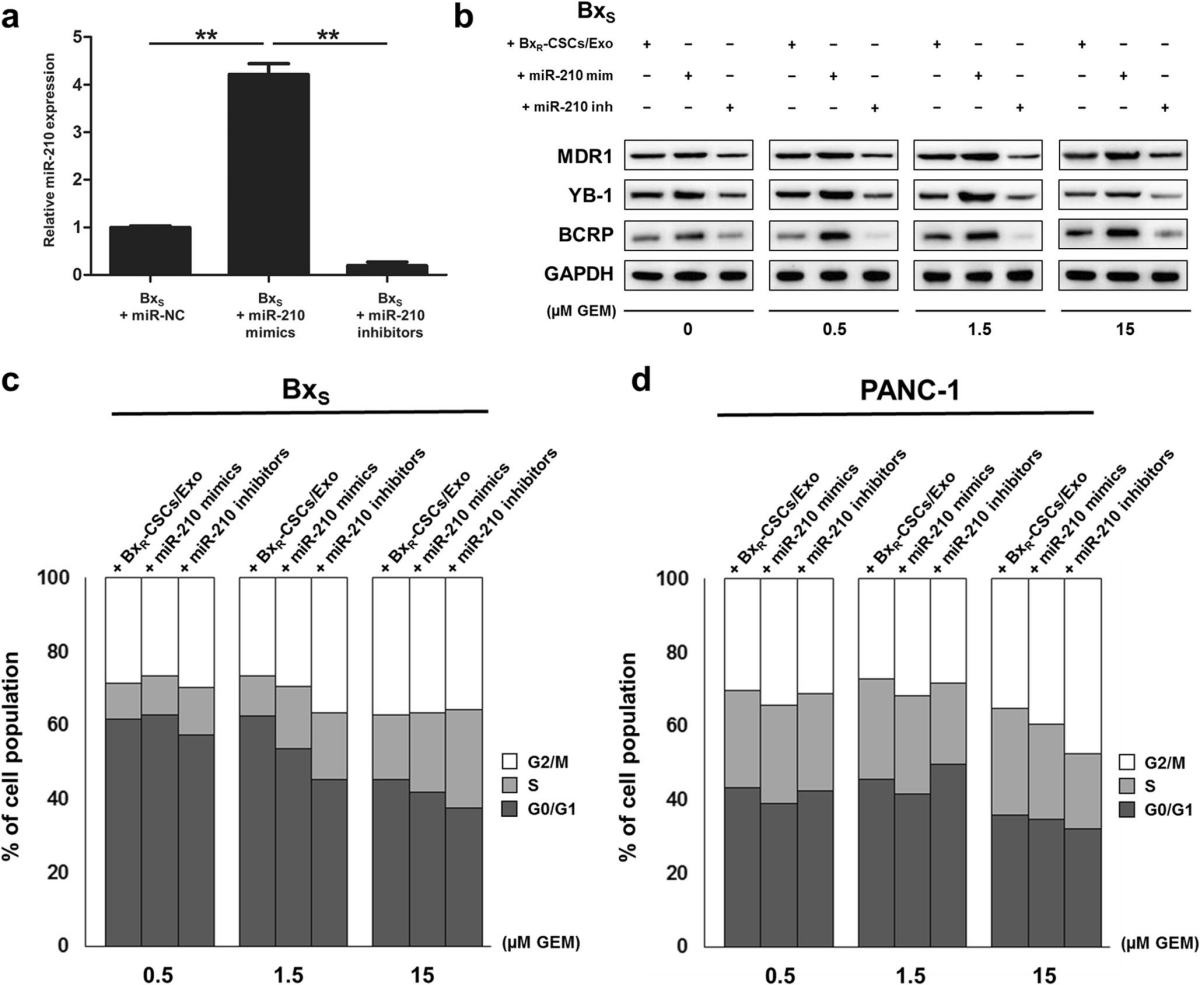
miR-210 mediates resistance of Bx_S_ cells to GEM. (**a**) Bx_S_ cells were transfected with miR-210 mimics or inhibitors after which miR-210 expression was detected by qRT-PCR. Statistical analysis was carried out using ANOVA. Data are presented as the mean ± SD (n = 3), ^**^p < 0.01. (**b**) Western blot analysis of drug resistance markers MDR1, YB-1, and BCRP in Bx_S_ cells after culture with Bx_R_-CSCs/Exo or transfection with miR-210 mimics/inhibitors, exposed to GEM at various concentrations (0 to 15 μM) for 48 h. Flow cytometric analysis of cell cycle progression in (**c**) Bx_S_ or (**d**) PANC-1 cells after culture with Bx_R_-CSCs/Exo or transfection with miR-210 mimics/inhibitors, exposed to GEM at various concentrations (0 to 15 μM) for 48 h

To determine whether miR-210 was involved in regulating GEM resistance, Bx_S_ cells were directly transfected with miR-210 mimics or inhibitors, followed by exposure to GEM. The successful transfection of miR-210 mimics or inhibitors was validated by qRT-PCR, showing that miR-210 was highly expressed after transfection of the mimics but only minimally expressed in case of the inhibitor or negative control (Fig. 5a). After exposure to GEM, the drug resistance-related proteins MDR1, YB-1 and BCRP were upregulated in cells subjected to Bx_R_-CSCs/Exo treatment or transfected with miR-210, compared to those in cells transfected with miR-210 inhibitors (Fig. 5b). In addition, the drug-resistant effect of miR-210 mimics seemed to be stronger than that of Bx_R_-CSCs/Exo.

**Fig. 6:**
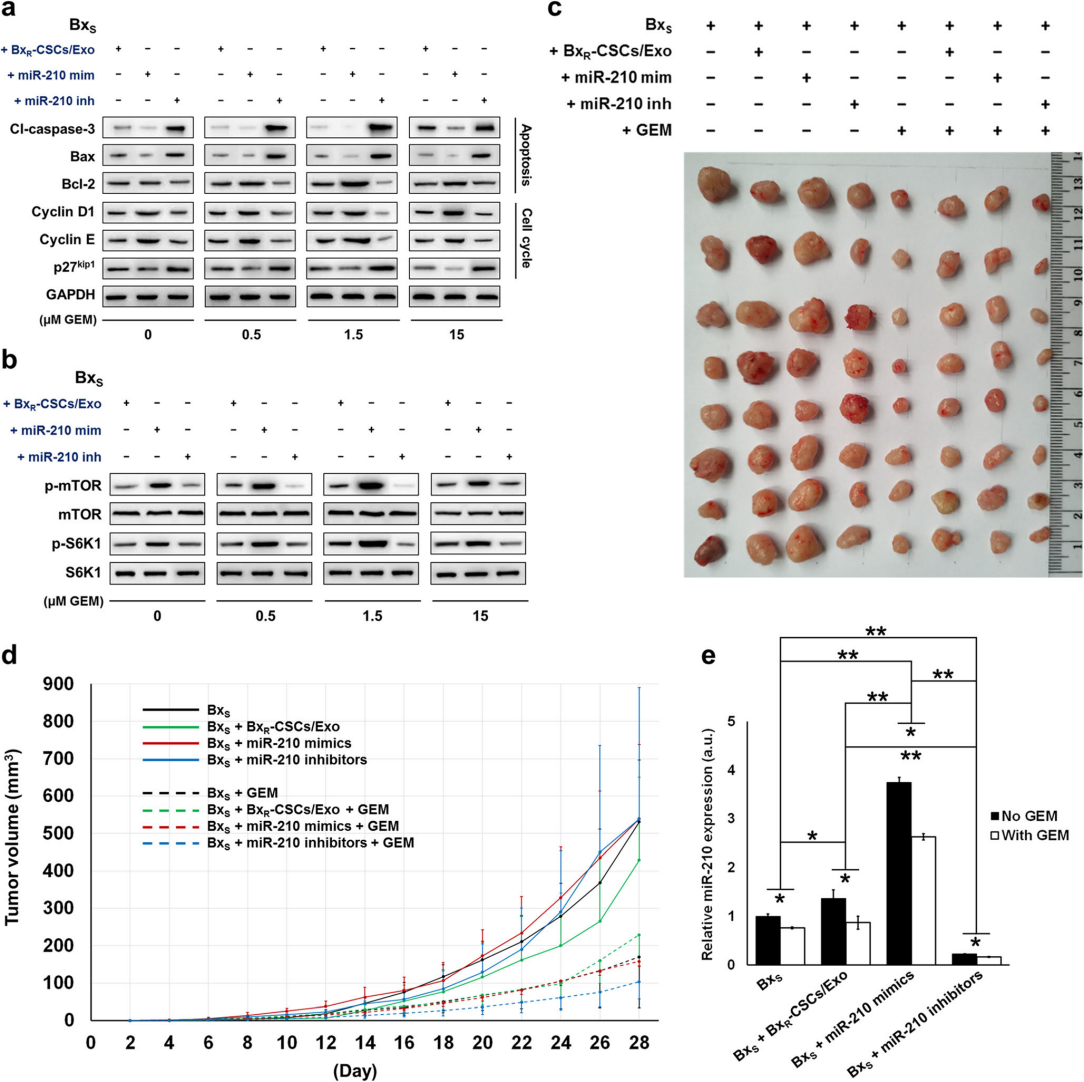
miR-210 activates mTOR signaling and promotes tumor growth in a nude mouse model. Western blot analysis of proteins associated with (**a**) apoptosis (cleaved caspase-3, Bax, and Bcl-2) and cell cycle progression (cyclin D1, cyclin E, and p27^kip1^) in Bx_S_ cells cultured with Bx_R_-CSCs/Exo or transfected with miR-210 mimics/inhibitors, exposed to GEM at various concentrations (0 to 15 μM) for 48 h. (**b**) Western blot analysis of mTOR and S6K1 phosphorylation (relative to total protein level) in Bx_S_ cells cultured with Bx_R_-CSCs/Exo or transfected with miR-210 mimics/inhibitors, exposed to GEM at various concentrations (0 to 15 μM) for 48 h. (**c**) Representative images of tumor growth and (**d**) corresponding tumor volumes 28 days after injection of Bx_R_-CSCs/Exo, miR-210 mimics, or miR-210 inhibitors in the presence or absence of GEM. (**e**) Expression of miR-210 in tumor tissues on day 28. Statistical analysis was carried out using ANOVA. Data are presented as the mean ± SD (n = 3), ^*^p < 0.05 and ^**^p < 0.01

To further assess the role of miR-210 delivered by exosomes in the proliferation and apoptosis of pancreatic cancer cells, transfected Bx_S_ and PANC-1 cells were exposed to GEM at increasing concentrations. Subsequent flow cytometry revealed that transfection with miR-210 inhibitors induced cell cycle arrest at the G2/M phase in a GEM dose-dependent manner in both Bx_S_ and PANC-1 cells, while no change in cell cycle distribution was observed after miR-210 mimic transfection and Bx_R_-CSCs/Exo treatment (Fig. 5c and d, Supplementary Fig. S7 and S8). In addition, overexpression of miR-210 inhibited GEM-induced apoptosis in both cell types, consistent with the effect of Bx_R_-CSCs/Exo (Table 2, Supplementary Fig. S9 and S10). These results were further validated by Western blot analysis of proteins associated with cell cycle progression (i.e., cyclin D1, cyclin E1 and p27^kip1^) and apoptosis (i.e., pro-apoptotic cleaved caspase-3 and Bax and anti-apoptotic Bcl-2) (Fig. 6a).

**Table 2:** Apoptosis of Bx_S_ and PANC-1 cells after treatment with Bx_R_-CSCs/Exo, miR-210 mimics, or miR-210 inhibitors, and exposure to GEM at various concentrations

		Bx_S_			PANC-1		
μM GEM		+ Bx_R_-CSCs /Exo	+ miR-210 mimics	+ miR-210 inhibitors	+ Bx_R_-CSCs /Exo	+ miR-210 mimics	+ miR-210 inhibitors
0.5	Early (%)	2.25	3.00	4.60	0.76	0.54	0.22
	Late (%)	6.67	8.12	21.54	10.63	9.69	34.88
	Total (%)	8.92	11.12	26.14	11.39	10.23	35.10
1.5	Early (%)	2.41	3.78	3.87	0.80	0.87	0.49
	Late (%)	9.21	11.67	31.67	10.95	10.94	37.19
	Total (%)	11.62	15.45	35.54	11.75	11.81	37.68
15	Early (%)	3.35	3.58	3.14	0.64	0.29	0.36
	Late (%)	10.60	13.55	37.42	11.98	15.70	64.06
	Total (%)	13.95	17.13	40.56	12.62	15.99	64.42

### miR-210 activates mTOR signaling and promotes tumor growth in vivo

The mTOR signaling pathway is known to play an important role in the proliferation, survival and motility of tumor cells [27, 28]. We evaluated the phosphorylation levels of mTOR and S6K1, a downstream target of mTOR, and found that they were significantly elevated in Bx_S_ cells treated with Bx_R_-CSCs/Exo or transfected with miR-210 mimics compared to those in cells transfected with miR-210 inhibitors (Fig. 6b). In addition, the administration of miR-210 inhibitors restricted tumor growth in mice compared to the administration of Bx_R_-CSCs/Exo or miR-210 mimics (Fig. 6c). When GEM was additionally administered, it effectively reduced the tumor volumes compared to those in the corresponding groups without GEM treatment (Fig. 6d). Conversely, mice treated with miR-210 inhibitors showed a more prominent reduction in tumor volume than those treated with Bx_R_-CSCs/Exo or miR-210 mimics, which was affected to a lesser extent by GEM due to the conferred drug resistance. Expression of miR-210 was also detected in tumor tissues (Fig. 6e), and mice injected with Bx_R_-CSCs/Exo showed higher levels of miR-210 than non-treated mice. Moreover, miR-210 mimics upregulated miR-210 levels in tumor tissues while the inhibitors suppressed miR-210 expression, as anticipated. In all cases, the administration of GEM effectively reduced miR-210 expression in the extracted tumor tissues. These data suggest that miR-210 transferred by Bx_R_-CSCs/Exo mediates the resistance of tumor cells to GEM by triggering the mTOR signaling pathway.

## Discussion

GEM chemoresistance is an intractable clinical problem in the treatment of pancreatic cancer. A thorough understanding of the mechanisms underlying drug resistance may pave the way for improving the therapeutic efficacy of GEM. In the current study, we found that exosomes derived from Bx_R_-CSCs can mediate the horizontal transfer of drug-resistant traits from Bx_R_ CSCs to Bx_S_ and PANC-1 human pancreatic cancer cells through the delivery of miR-210.

CSCs have been suggested as both the seeds of primary cancer development and the roots of chemo- and radiotherapy resistance [29, 30]. Multiple mechanisms have been found to be involved in CSC-mediated drug resistance, including decreased cell cycle progression, increased drug efflux, enhanced DNA repair efficiency, elevated anti-apoptotic capacity, and overexpression of detoxification enzymes [31]. Here, we isolated CSCs from Bx_S_ and Bx_R_ cells and investigated the sensitivity of these CSCs to GEM. CSCs derived from Bx_R_ cells exhibited a greater resistance to GEM compared to those derived from Bx_S_ cells. In addition, the expression of the drug resistance-related proteins MDR, YB-1 and BCRP decreased in Bx_S_-CSCs in a GEM dose-dependent manner, while this expression remained unchanged in Bx_R_-CSCs. These results indicate that Bx_R_-CSCs exhibit resistance to GEM, which may be the major cause of the resistance of Bx_R_ cells to GEM.

Interactions of CSCs with their microenvironment are critical for drug resistance [32]. Recently, exosomes have been investigated as novel messengers in cell-to-cell communication, and it was reported that exosomes mediated the horizontal transfer of drug-resistant traits from imatinib-resistant chronic myeloid leukemia cells to imatinib-sensitive cells [21]. CSCs secrete exosomes to interact with both surrounding cancer cells and stromal cells [9]. Here, we isolated exosomes from the culture medium of CSCs derived from Bx_R_ or Bx_S_ cells. It has been reported that exosomes exert effects on recipient cells via endocytosis and release of their cargo [33–35]. Our data indicate that exosomes derived from Bx_R_-CSCs enhance the resistance of Bx_S_ and PANC-1 cells to GEM. When Bx_S_ or PANC-1 cells were cultured with Bx_R_-CSCs/Exo, they exhibited a stronger resistance to GEM. In addition, Bx_R_-CSCs/Exo slowed down GEM-induced cell cycle arrest, antagonized GEM-induced apoptosis, and promoted tube formation and cell migration.

Enhanced resistance of Bx_S_ and PANC-1 cells to GEM via Bx_R_-CSCs/Exo may be associated with the release of their cargo. Emerging evidence indicates that exosomes can transfer proteins, DNA, mRNAs, miRNAs and lncRNAs from donor cells to recipient cells, thereby modulating their biological behavior [25, 26]. CSC-derived exosomes are major transport vehicles for miRNAs and play important roles in cell-to-cell communication [36]. Additional evidence indicates that exosomes derived from drug-resistant cancer cells can transmit chemoresistance to drug-sensitive cancer cells via the transfer of miRNAs [21, 26], in particular miR-145, miR-210, miR-509 and miR-1243, which are known to be associated with CSC-mediated chemoresistance and/or radio-resistance [37, 38]. Here, we detected the above-mentioned drug resistance-related miRNAs in Bx_S_-CSCs, Bx_R_-CSCs and CSC-derived exosomes using qRT-PCR. The expression of miR-210 in Bx_R_-CSCs was significantly increased in a GEM dose-dependent manner and was highly expressed in Bx_R_-CSCs/Exo. In addition, treatment with Bx_R_-CSCs/Exo resulted in increased expression of miR-210 in Bx_S_ and PANC-1 cells exposed to GEM. Previously, MiR-210 has been shown to enhance radio-resistance in human lung cancer cells [39]. Taken together, we conclude that exosomes may mediate a horizontal transfer of miR-210 between Bx_R_ and Bx_S_/PANC-1 cells to confer drug resistance.

MiR-210 has been found to be abundantly expressed in multiple malignant tumors, and a number of miR-210 target genes has been reported to participate in cell cycle progression, DNA repair, vascular generation and CSC survival [40]. Here, we found that overexpression of miR-210 slowed down cell cycle arrest at the G2/M phase, inhibited GEM-induced apoptosis, and promoted Bx_S_ tumor growth in nude mice, as opposed to the effects of miR-210 silencing. Interestingly, the changes induced by miR-210 overexpression were consistent with the intervention of Bx_R_-CSCs/Exo. In addition, the phosphorylation levels of mTOR and S6K1 were significantly elevated in Bx_S_ cells treated with Bx_R_-CSCs/Exo or transfected with miR-210 mimics compared to those in cells transfected with miR-210 inhibitors. Previously, potential targets of miR-210 associated with mTOR signaling have been identified. Specifically, miR-210 plays protective roles against hypoxia-induced injury by targeting Bcl-2 adenovirus E1B 19 kDa-interacting protein 3 (BNIP3), which in turn leads to PI3K/AKT/mTOR pathway activation [41, 42]. Similarly, BTG3 has been identified as a target of miR-210 in neural stem cells, and overexpression of miR-210 negatively regulated the expression of BTG3 and enhanced PI3K/AKT/mTOR pathway activation [43]. Our findings are in agreement with these reports and indicate that miR-210 promoted the proliferation and inhibited apoptosis in Bx_S_ cells by activating the mTOR signaling pathway.

In summary, we conclude that exosomes derived from Bx_R_-CSCs can mediate a horizontal transfer of drug-resistant traits by delivering miR-210. This finding offers an explanation for GEM treatment failure in pancreatic cancer and may be instrumental for the design of novel targeted strategies for the treatment of advanced pancreatic cancer.

## Electronic supplementary material

Fig. S1 Representative images of sphere formation from Bx_S_-CSCs and Bx_R_-CSCs. Scale bar = 100 μm. (PNG 3.78 mb)

High Resolution Image (TIFF 3.78 MB)

Fig. S2 Relative viability of Bx_S_ and PANC-1 cells after treatment with Bx_S_-CSCs/Exo or Bx_R_-CSCs/Exo at various concentrations of GEM (from 0 to 800 μM), measured by MTT assay. (PNG 438 kb)

High Resolution Image (TIFF 423 kb)

Fig. S3 Flow cytometric analysis of cell cycle progression in Bx_S_ cells after treatment with Bx_S_-CSCs/Exo or Bx_R_-CSCs/Exo at various concentrations of GEM (from 0.5 to 15 μM). (PNG 927 kb)

High Resolution Image (TIFF 389 kb)

Fig. S4 Flow cytometric analysis of cell cycle progression in PANC-1 cells after treatment with Bx_S_-CSCs/Exo or Bx_R_-CSCs/Exo at various concentrations of GEM (from 0.5 to 15 μM). (PNG 1.11 mb)

High Resolution Image (TIFF 573 kb)

Fig. S5 Flow cytometric analysis of apoptosis in Bx_S_ cells after treatment with Bx_S_-CSCs/Exo or Bx_R_-CSCs/Exo at various concentrations of GEM (from 0.5 to 15 μM). Numbers in the B4 and B2 quadrants represent the percentage of early and late apoptotic cells, respectively. (PNG 1.18 mb)

High Resolution Image (TIFF 452 kb)

Fig. S6 Flow cytometric analysis of apoptosis in PANC-1 cells after treatment with Bx_S_-CSCs/Exo or Bx_R_-CSCs/Exo at various concentrations of GEM (from 0.5 to 15 μM). Numbers in the B4 and B2 quadrants represent the percentage of early and late apoptotic cells, respectively. (PNG 1.34 mb)

High Resolution Image (TIFF 543 kb)

Fig. S7 Flow cytometric analysis of cell cycle progression in Bx_S_ cells after treatment with Bx_R_-CSCs/Exo, miR-210 mimics, or miR-210 inhibitors at various concentrations of GEM (from 0.5 to 15 μM). (PNG 1.23 mb)

High Resolution Image (TIFF 509 kb)

Fig. S8 Flow cytometric analysis of cell cycle progression in PANC-1 cells after treatment with Bx_R_-CSCs/Exo, miR-210 mimics, or miR-210 inhibitors at various concentrations of GEM (from 0.5 to 15 μM). (PNG 1.40 mb)

High Resolution Image (TIFF 743 kb)

Fig. S9 Flow cytometric analysis of apoptosis in Bx_S_ cells after treatment with Bx_R_-CSCs/Exo, miR-210 mimics, or miR-210 inhibitors at various concentrations of GEM (from 0.5 to 15 μM). Numbers in the B4 and B2 quadrants represent the percentage of early and late apoptotic cells, respectively. (PNG 1.40 mb)

High Resolution Image (TIFF 530 kb)

Fig. S10 Flow cytometric analysis of apoptosis in PANC-1 cells after treatment with Bx_R_-CSCs/Exo, miR-210 mimics, or miR-210 inhibitors at various concentrations of GEM (from 0.5 to 15 μM). Numbers in the B4 and B2 quadrants represent the percentage of early and late apoptotic cells, respectively. (PNG 1.38 mb)

High Resolution Image (TIFF 589 kb)
