# Supplementary figures and images for: Exosomes derived from cancer stem cells of gemcitabine-resistant pancreatic cancer cells enhance drug resistance by delivering miR-210
Source: Cell Oncol (Dordr). 2019 Nov 12;43(1):123–36. doi: 10.1007/s13402-019-00476-6 (PMC12990725; doi:10.1007/s13402-019-00476-6)

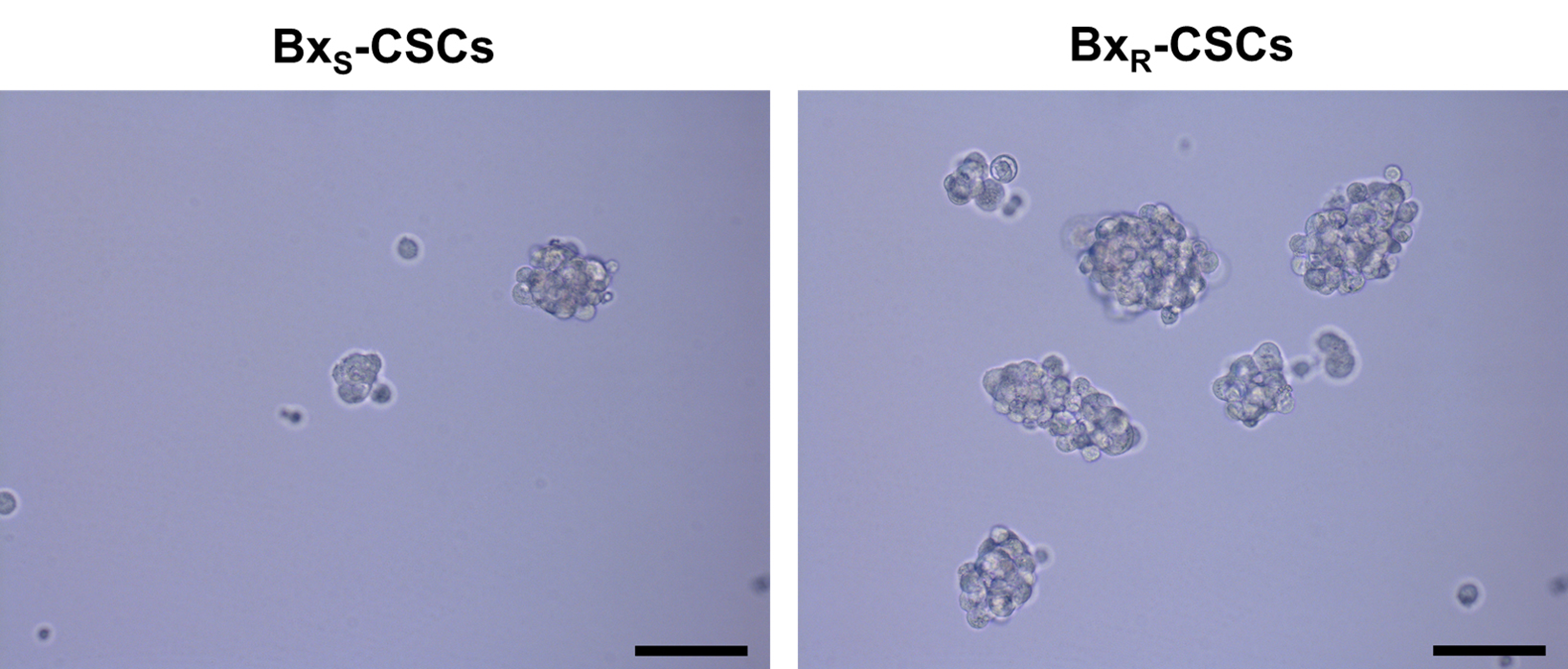

Supplement: Supplementary file 1 — Representative images of sphere formation from BxS-CSCs and BxR-CSCs. Scale bar = 100 μm. (PNG 3.78 mb) [file 13402_2019_476_Fig7_ESM.png]

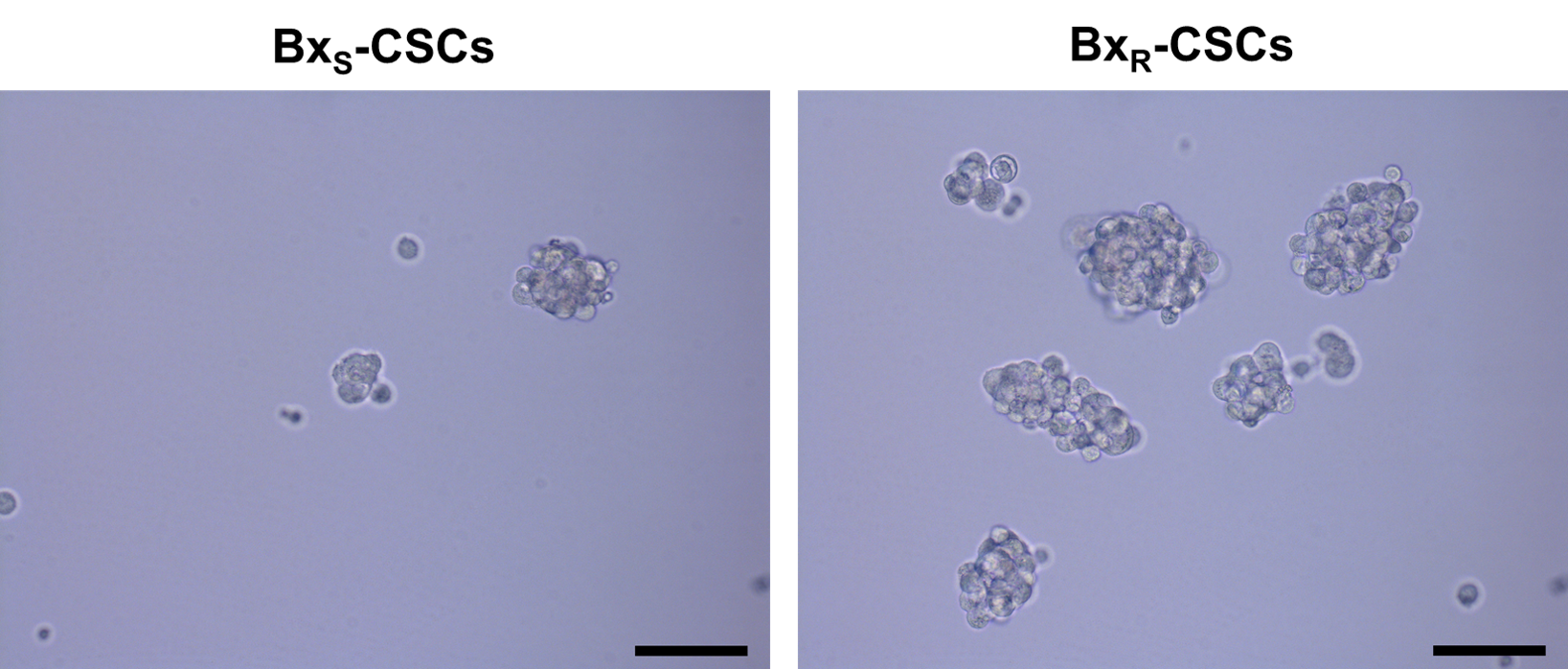

Supplement: Supplementary file 2 — High Resolution Image (TIFF 3.78 MB) [file 13402_2019_476_MOESM1_ESM.tif]

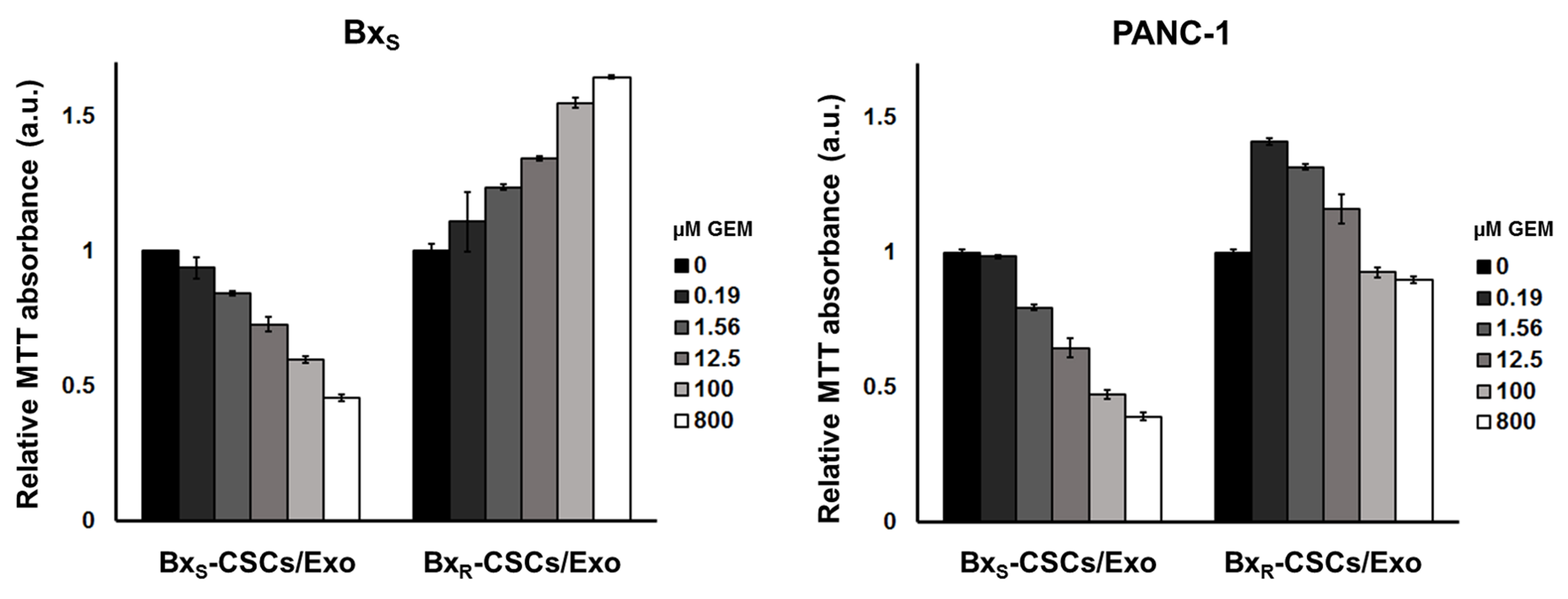

Supplement: Supplementary file 3 — Relative viability of BxS and PANC-1 cells after treatment with BxS-CSCs/Exo or BxR-CSCs/Exo at various concentrations of GEM (from 0 to 800 μM), measured by MTT assay. (PNG 438 kb) [file 13402_2019_476_Fig8_ESM.png]

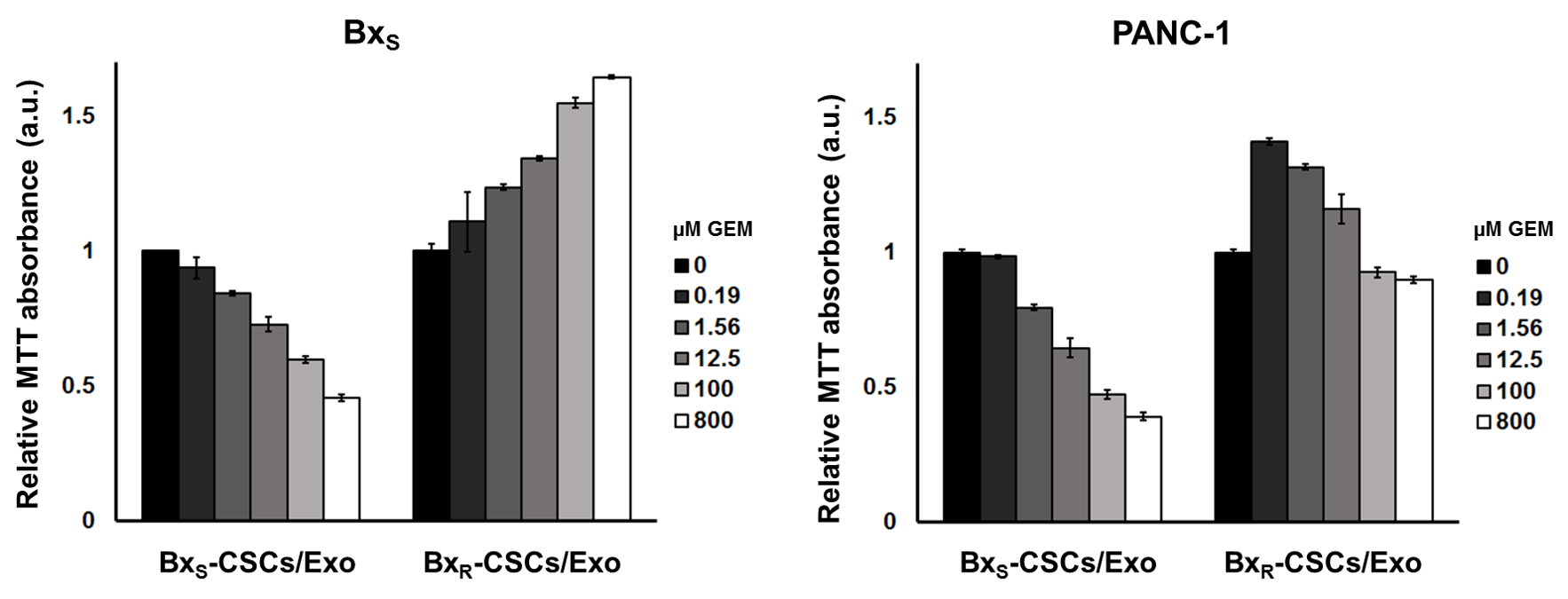

Supplement: Supplementary file 4 — High Resolution Image (TIFF 423 kb) [file 13402_2019_476_MOESM2_ESM.tif]

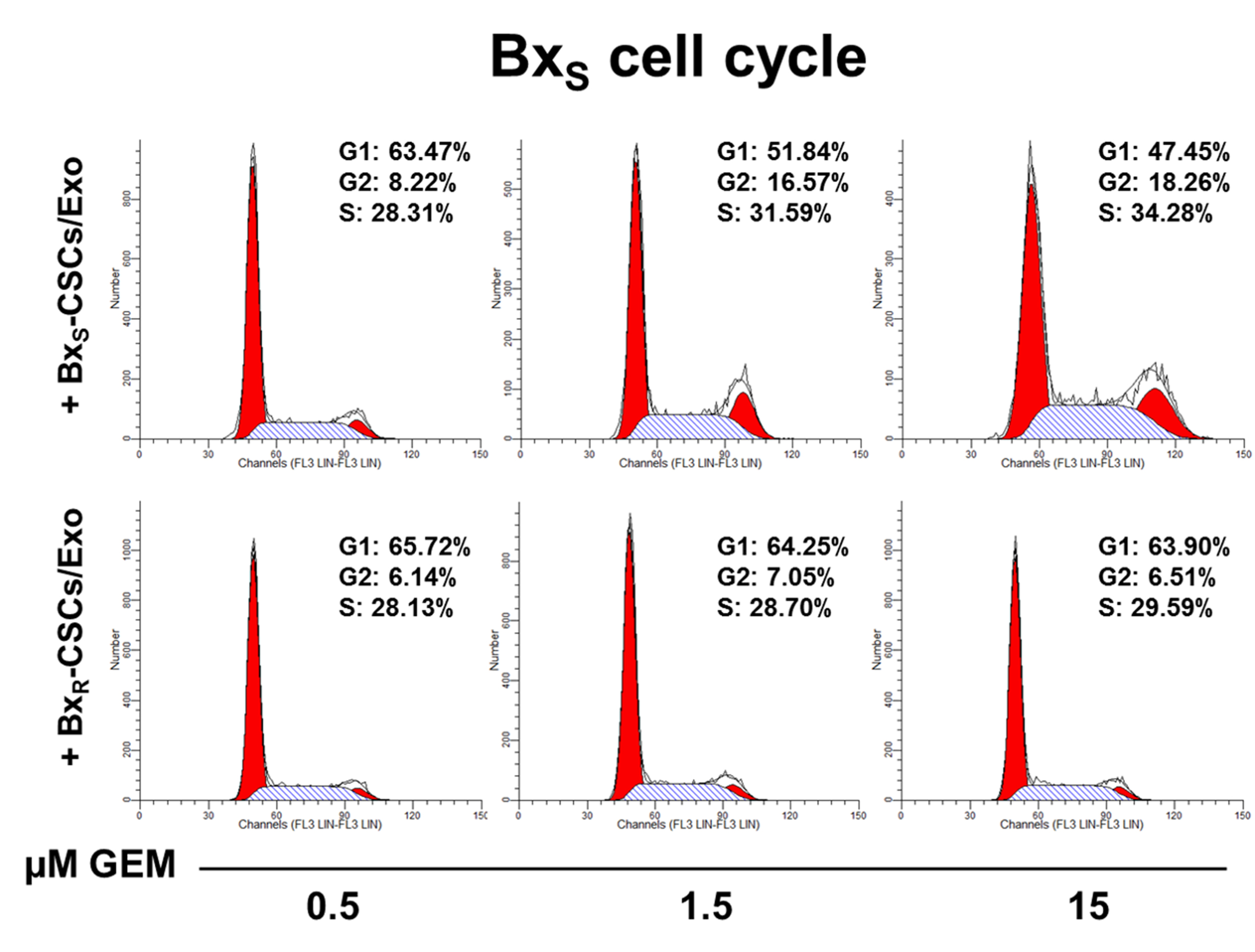

Supplement: Supplementary file 5 — Flow cytometric analysis of cell cycle progression in BxS cells after treatment with BxS-CSCs/Exo or BxR-CSCs/Exo at various concentrations of GEM (from 0.5 to 15 μM). (PNG 927 kb) [file 13402_2019_476_Fig9_ESM.png]

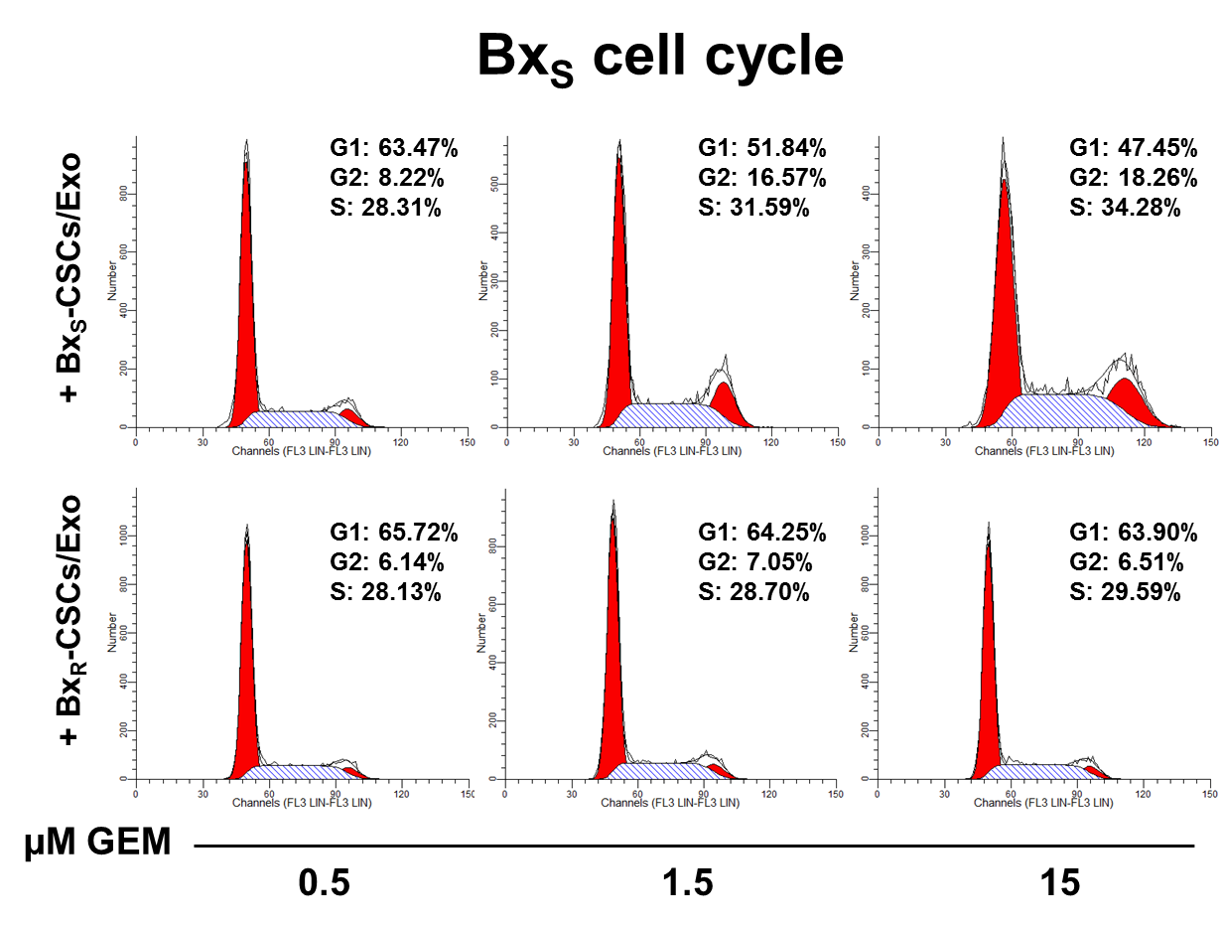

Supplement: Supplementary file 6 — High Resolution Image (TIFF 389 kb) [file 13402_2019_476_MOESM3_ESM.tif]

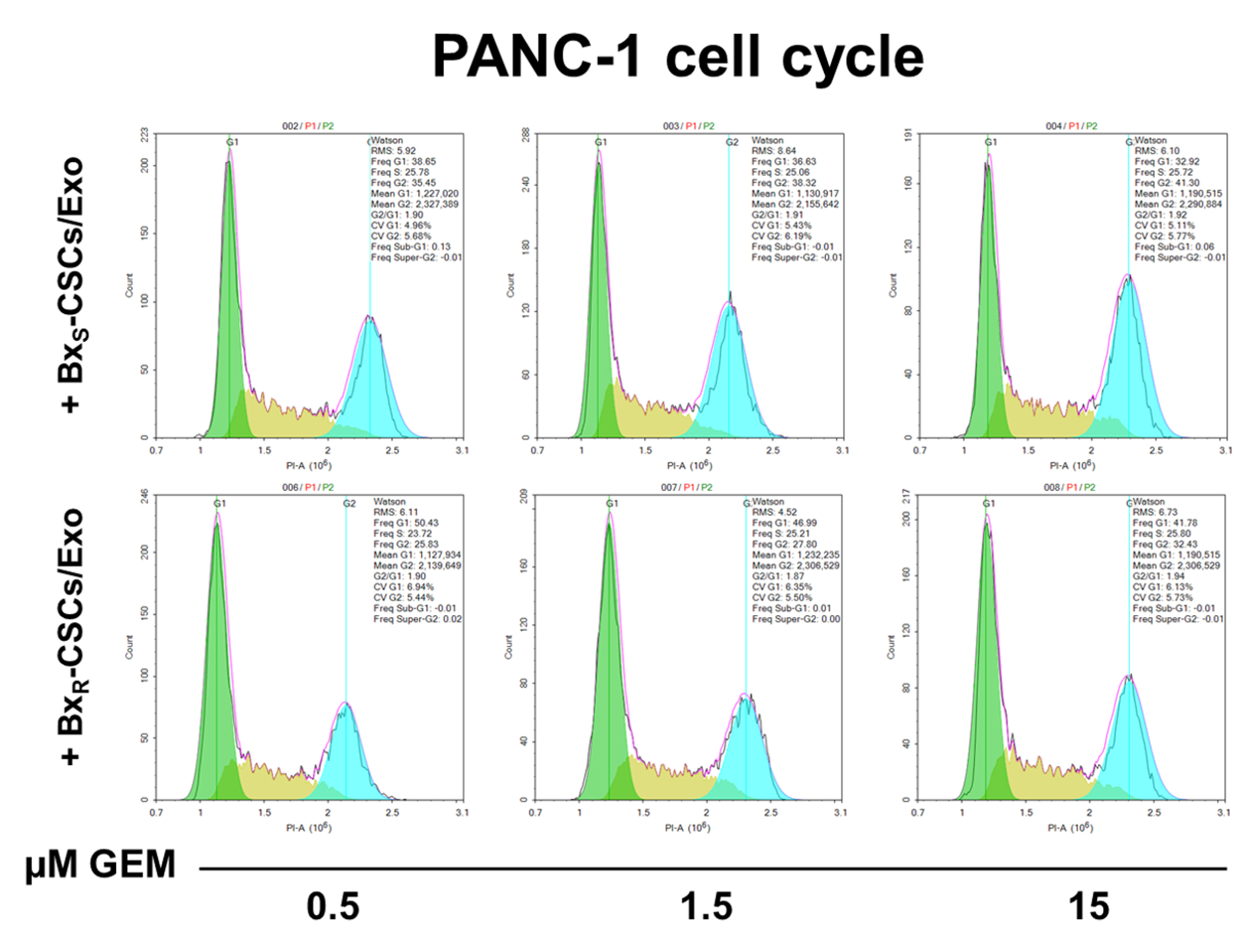

Supplement: Supplementary file 7 — Flow cytometric analysis of cell cycle progression in PANC-1 cells after treatment with BxS-CSCs/Exo or BxR-CSCs/Exo at various concentrations of GEM (from 0.5 to 15 μM). (PNG 1.11 mb) [file 13402_2019_476_Fig10_ESM.png]

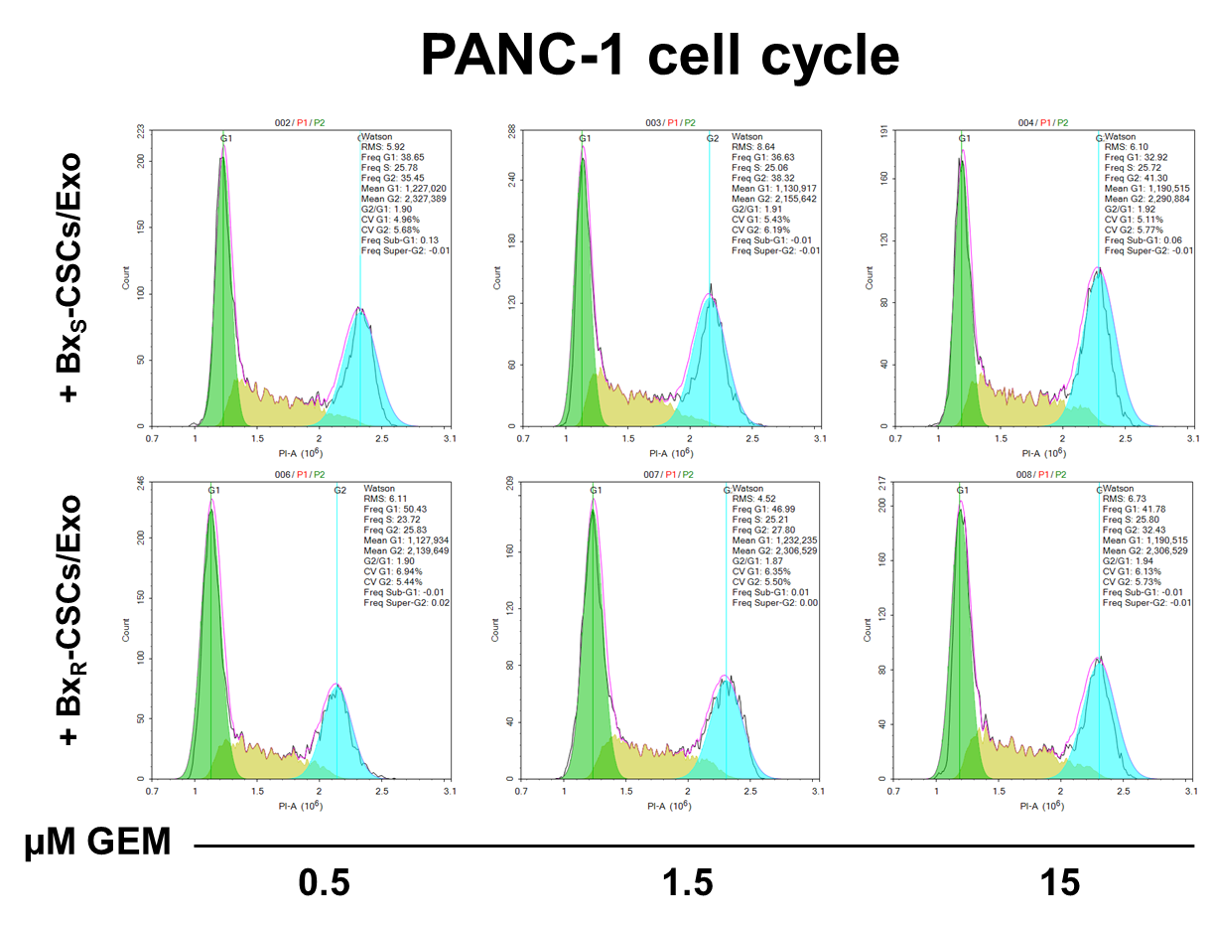

Supplement: Supplementary file 8 — High Resolution Image (TIFF 573 kb) [file 13402_2019_476_MOESM4_ESM.tif]

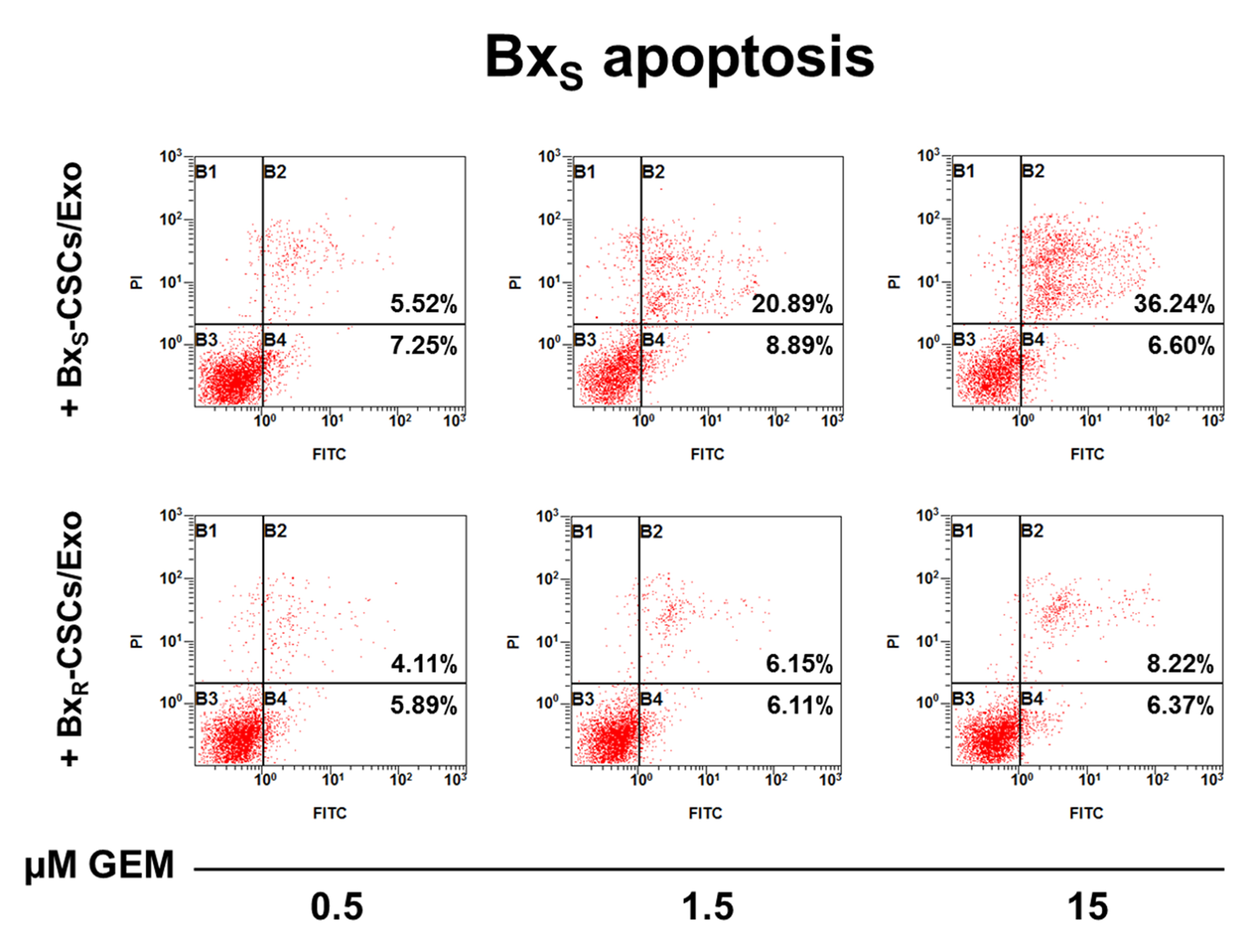

Supplement: Supplementary file 9 — Flow cytometric analysis of apoptosis in BxS cells after treatment with BxS-CSCs/Exo or BxR-CSCs/Exo at various concentrations of GEM (from 0.5 to 15 μM). Numbers in the B4 and B2 quadrants represent the percentage of early and late apoptotic cells, respectively. (PNG 1.18 mb) [file 13402_2019_476_Fig11_ESM.png]

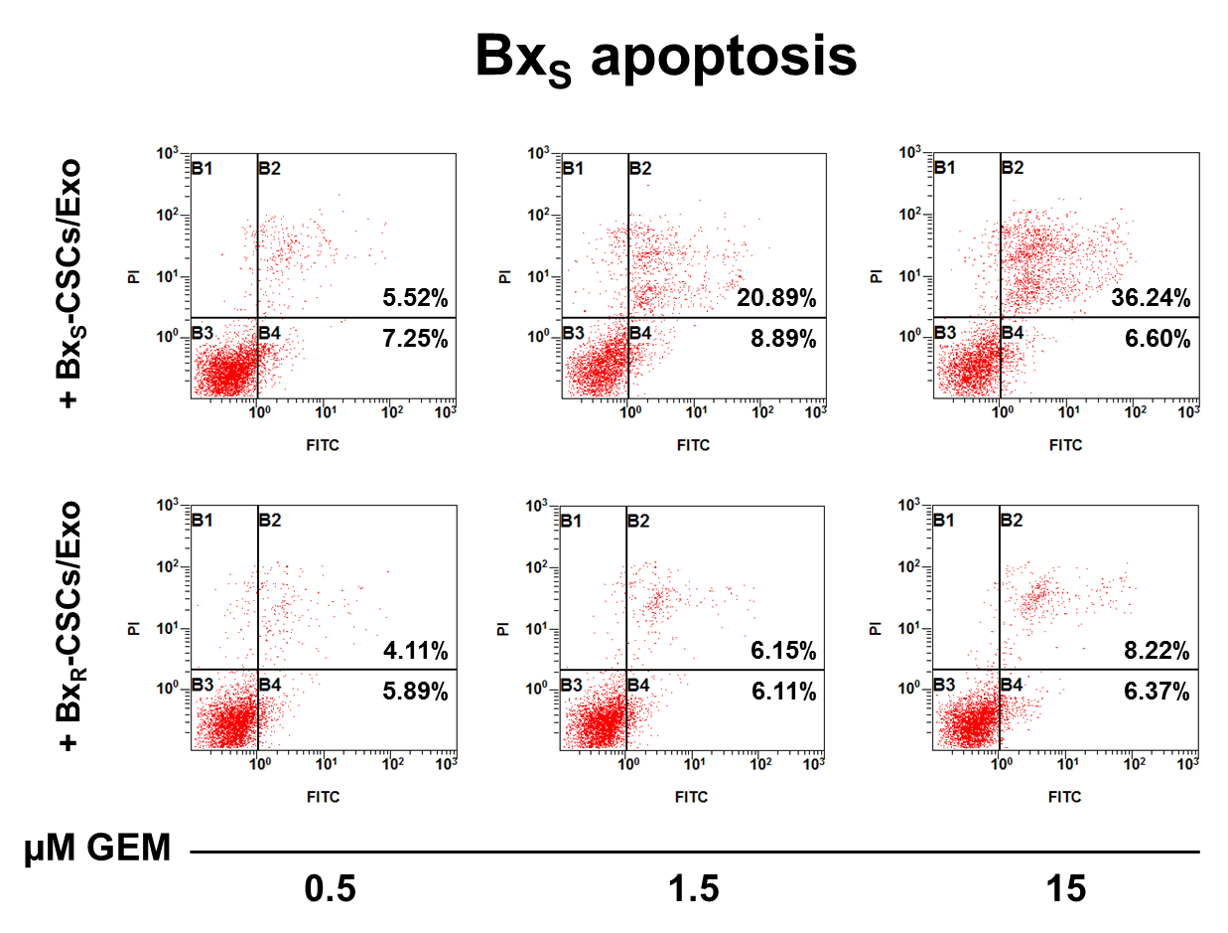

Supplement: Supplementary file 10 — High Resolution Image (TIFF 452 kb) [file 13402_2019_476_MOESM5_ESM.tif]

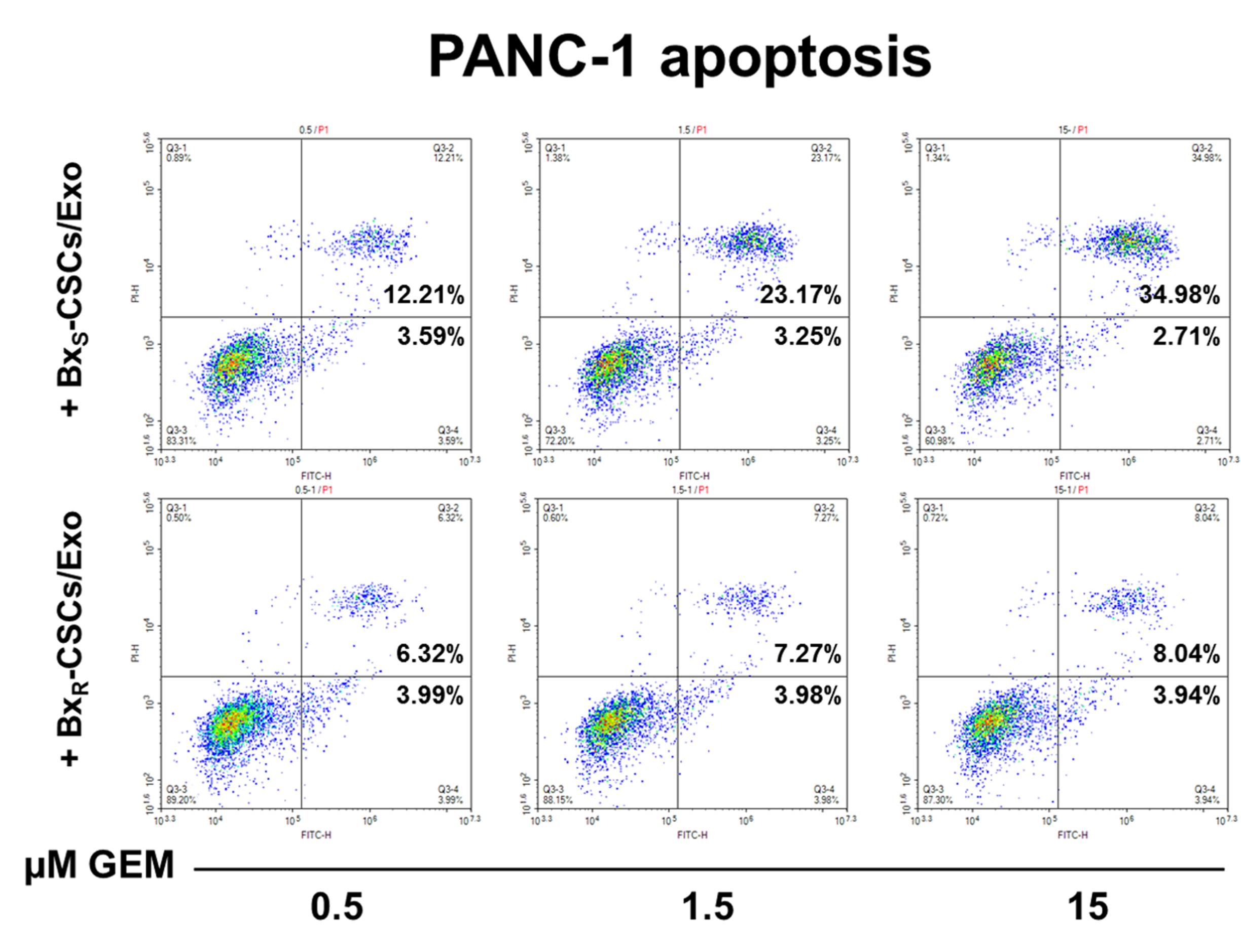

Supplement: Supplementary file 11 — Flow cytometric analysis of apoptosis in PANC-1 cells after treatment with BxS-CSCs/Exo or BxR-CSCs/Exo at various concentrations of GEM (from 0.5 to 15 μM). Numbers in the B4 and B2 quadrants represent the percentage of early and late apoptotic cells, respectively. (PNG 1.34 mb) [file 13402_2019_476_Fig12_ESM.png]

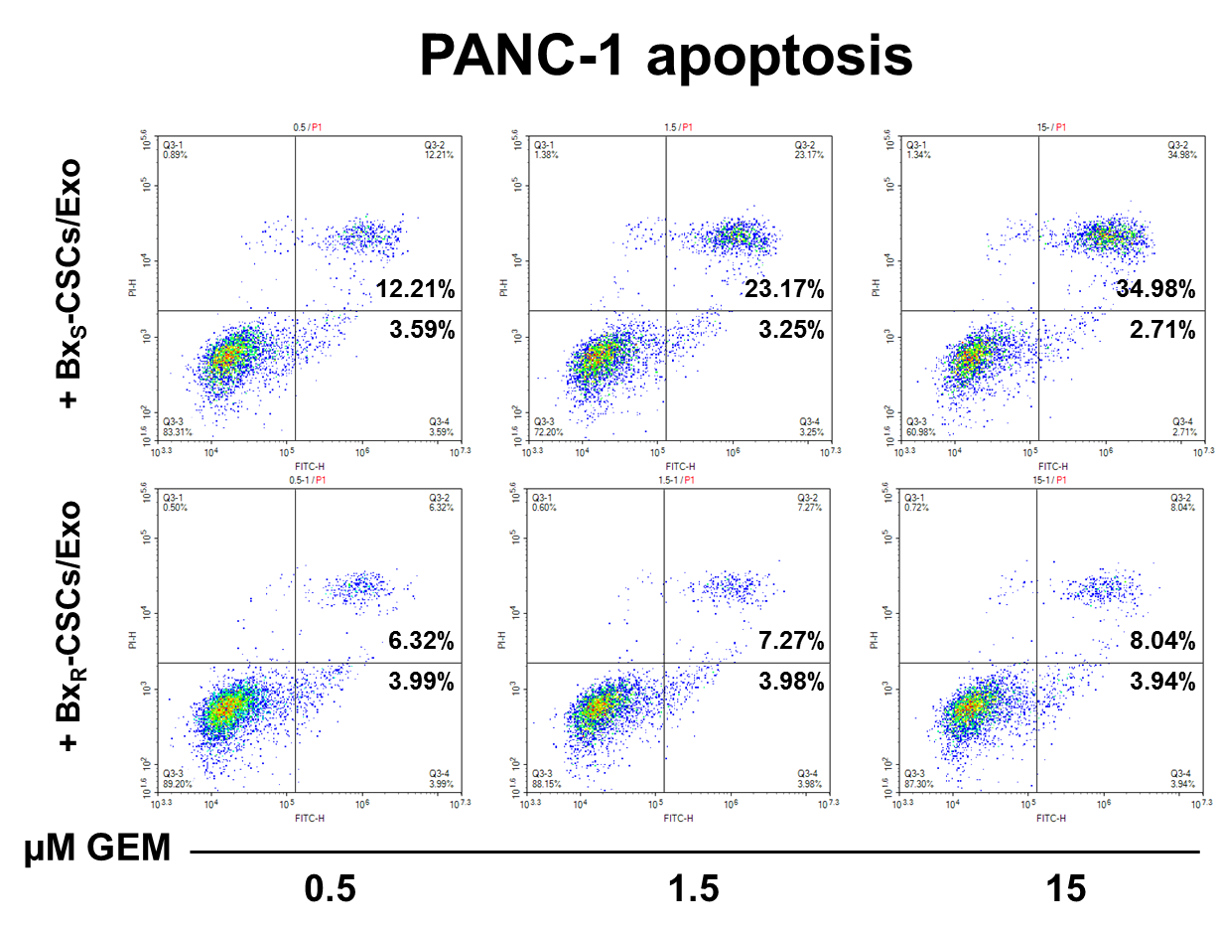

Supplement: Supplementary file 12 — High Resolution Image (TIFF 543 kb) [file 13402_2019_476_MOESM6_ESM.tif]

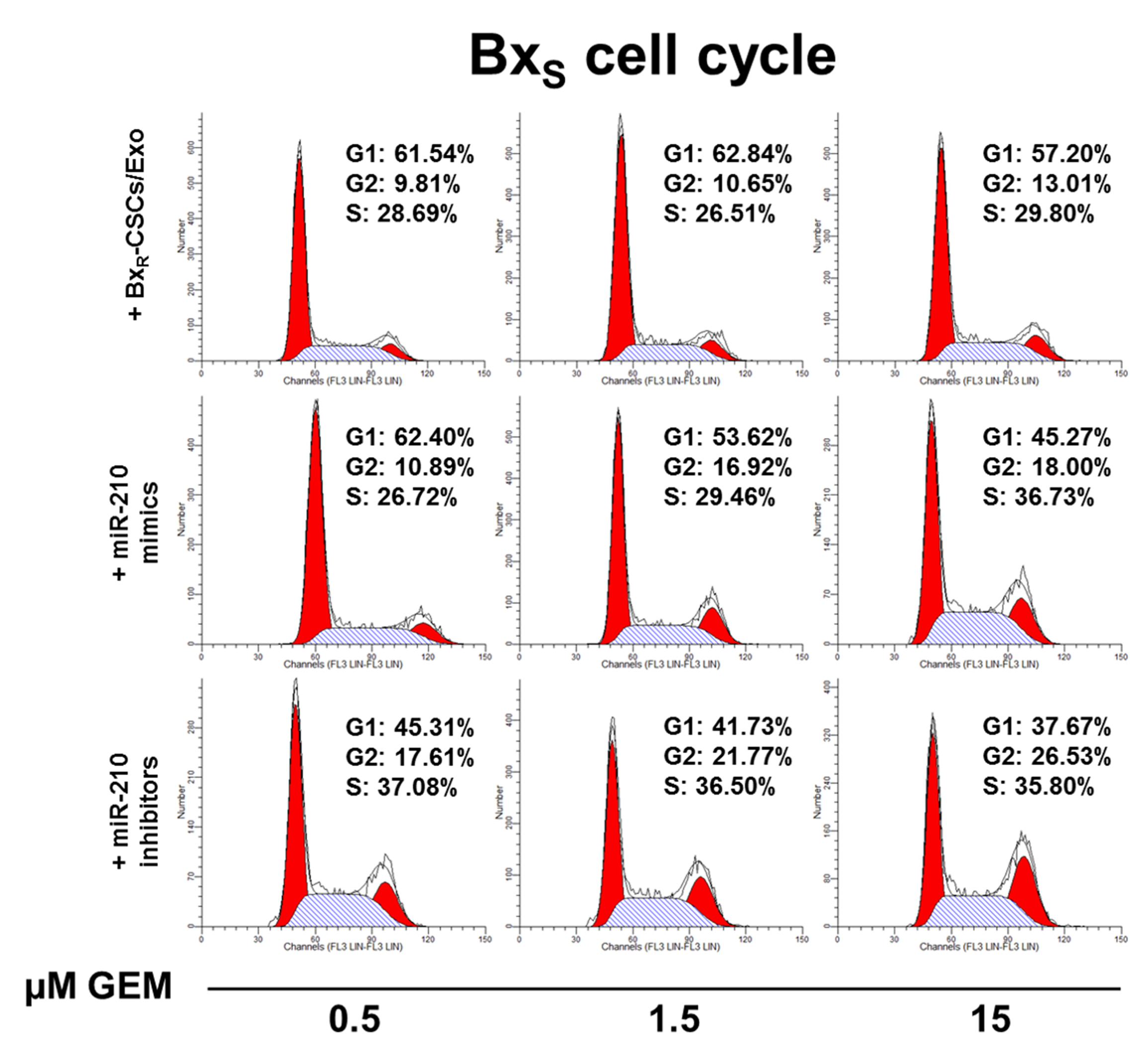

Supplement: Supplementary file 13 — Flow cytometric analysis of cell cycle progression in BxS cells after treatment with BxR-CSCs/Exo, miR-210 mimics, or miR-210 inhibitors at various concentrations of GEM (from 0.5 to 15 μM). (PNG 1.23 mb) [file 13402_2019_476_Fig13_ESM.png]

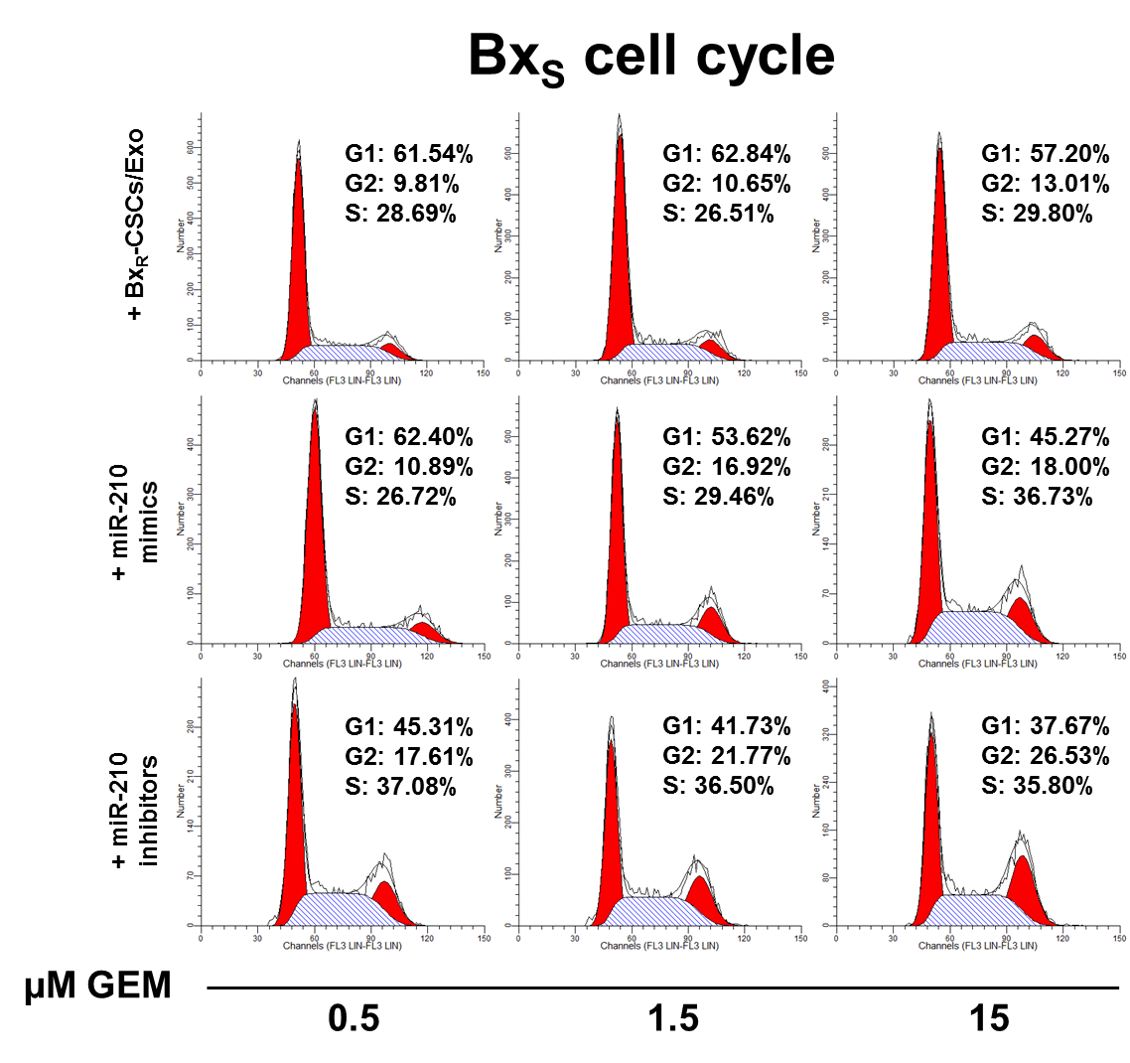

Supplement: Supplementary file 14 — High Resolution Image (TIFF 509 kb) [file 13402_2019_476_MOESM7_ESM.tif]

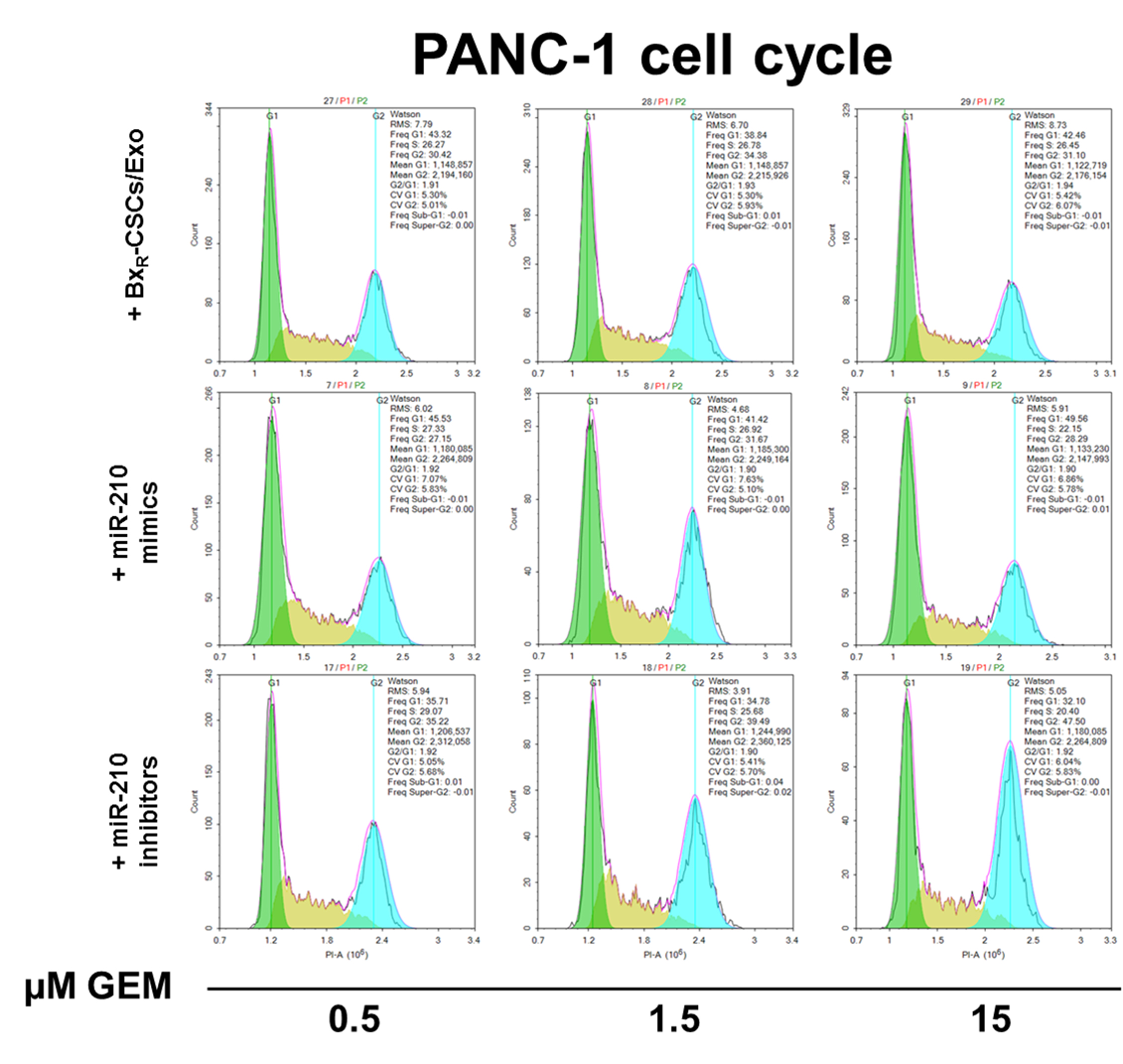

Supplement: Supplementary file 15 — Flow cytometric analysis of cell cycle progression in PANC-1 cells after treatment with BxR-CSCs/Exo, miR-210 mimics, or miR-210 inhibitors at various concentrations of GEM (from 0.5 to 15 μM). (PNG 1.40 mb) [file 13402_2019_476_Fig14_ESM.png]

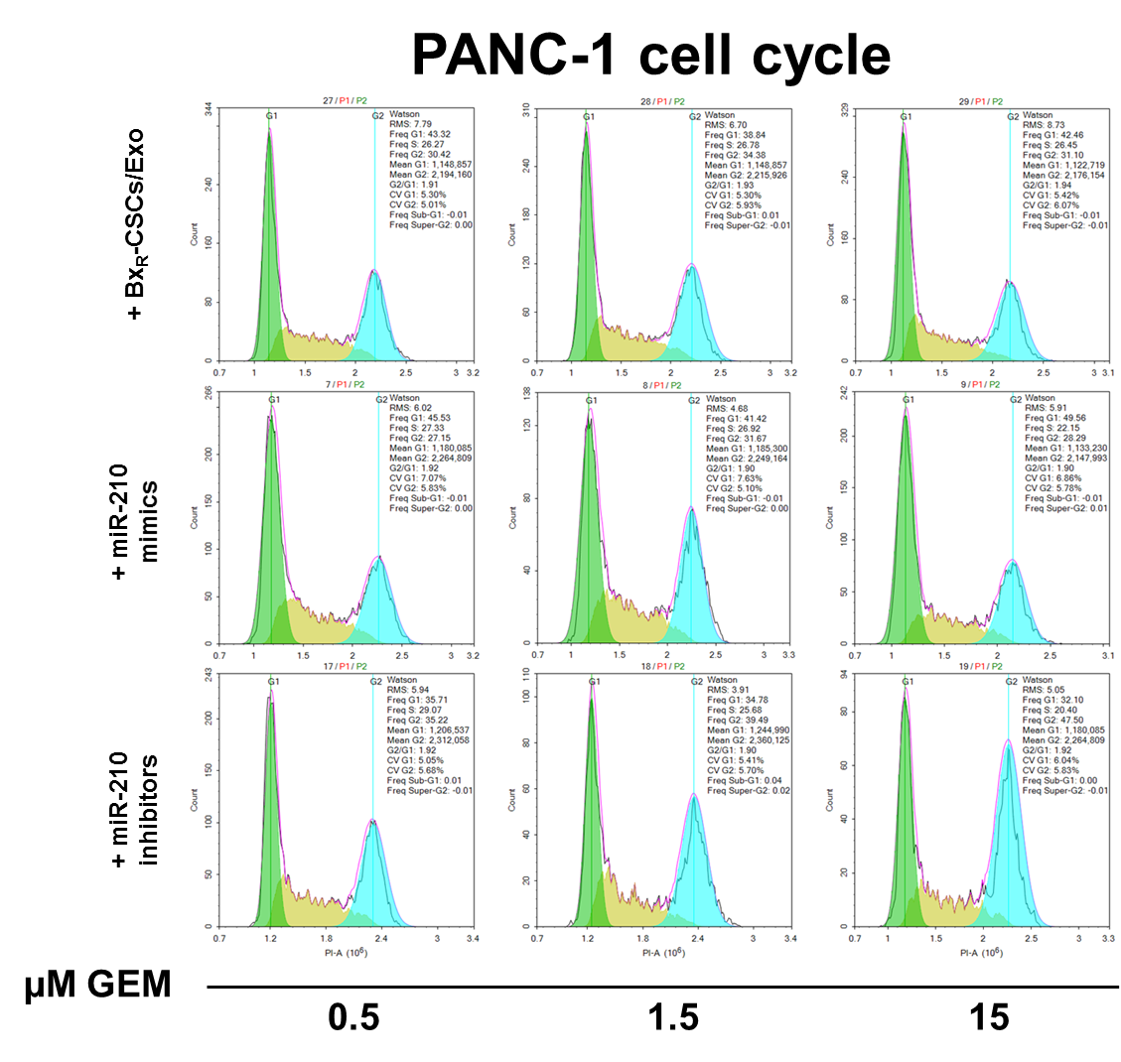

Supplement: Supplementary file 16 — High Resolution Image (TIFF 743 kb) [file 13402_2019_476_MOESM8_ESM.tif]

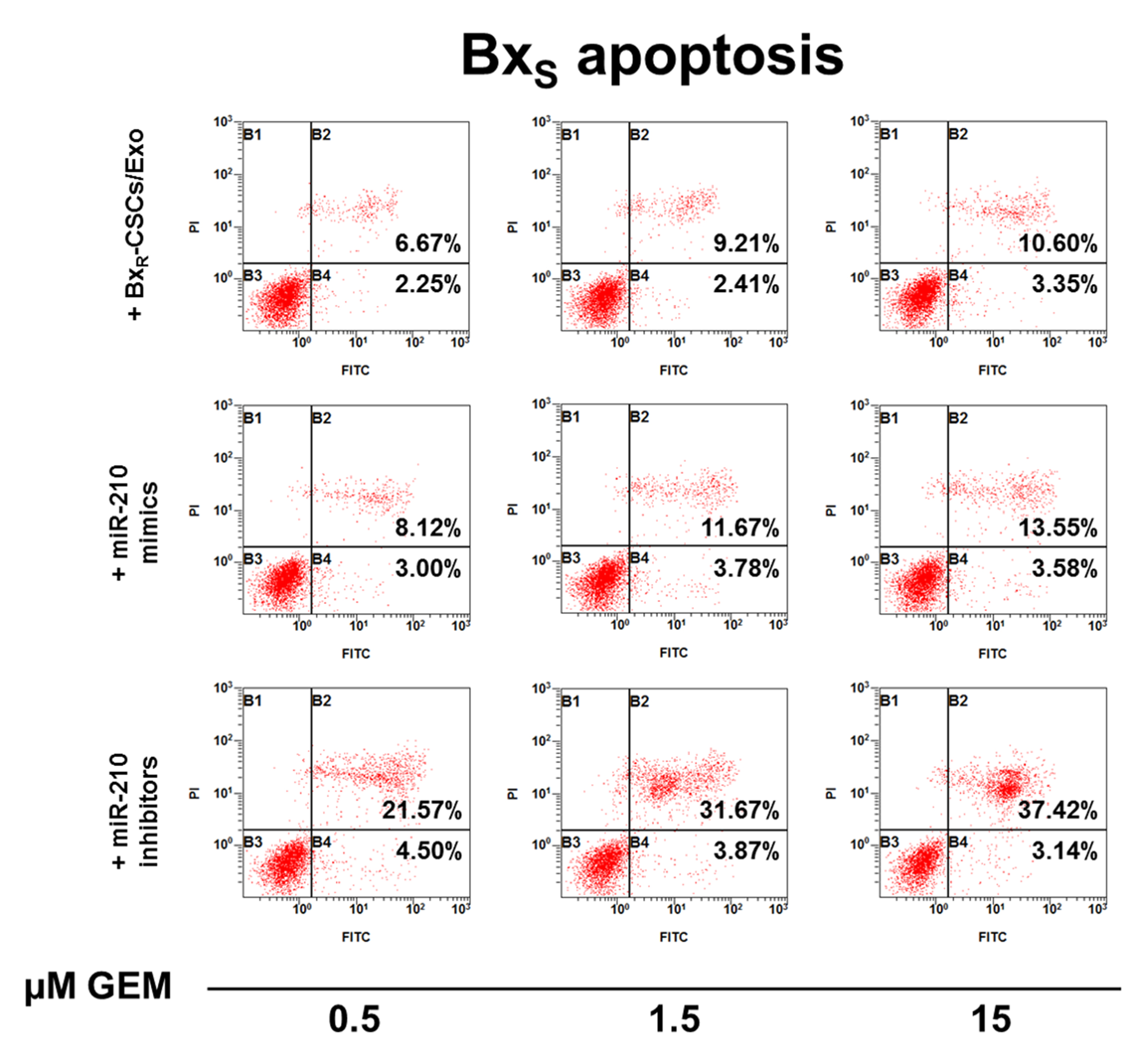

Supplement: Supplementary file 17 — Flow cytometric analysis of apoptosis in BxS cells after treatment with BxR-CSCs/Exo, miR-210 mimics, or miR-210 inhibitors at various concentrations of GEM (from 0.5 to 15 μM). Numbers in the B4 and B2 quadrants represent the percentage of early and late apoptotic cells, respectively. (PNG 1.40 mb) [file 13402_2019_476_Fig15_ESM.png]

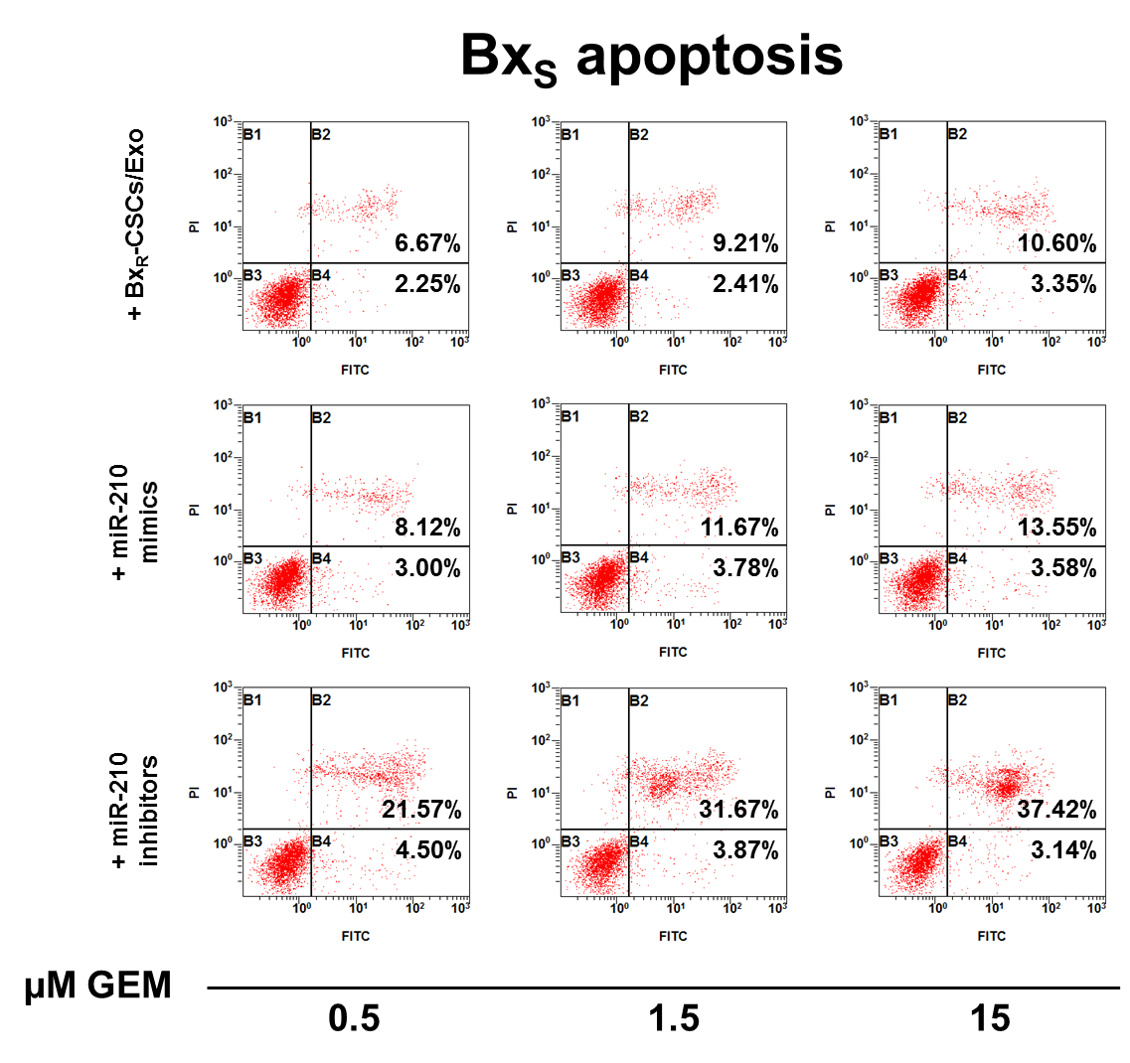

Supplement: Supplementary file 18 — High Resolution Image (TIFF 530 kb) [file 13402_2019_476_MOESM9_ESM.tif]

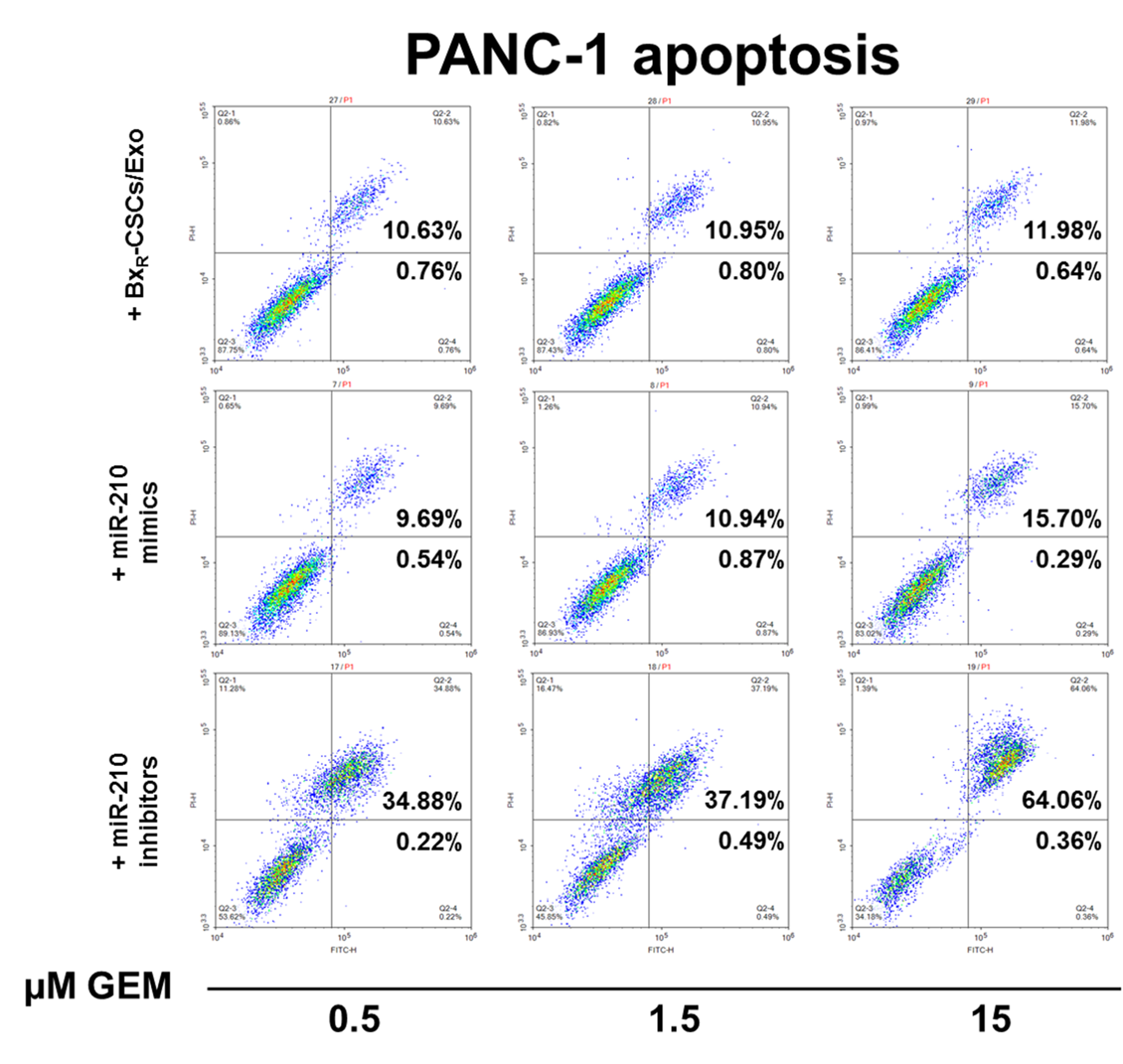

Supplement: Supplementary file 19 — Flow cytometric analysis of apoptosis in PANC-1 cells after treatment with BxR-CSCs/Exo, miR-210 mimics, or miR-210 inhibitors at various concentrations of GEM (from 0.5 to 15 μM). Numbers in the B4 and B2 quadrants represent the percentage of early and late apoptotic cells, respectively. (PNG 1.38 mb) [file 13402_2019_476_Fig16_ESM.png]

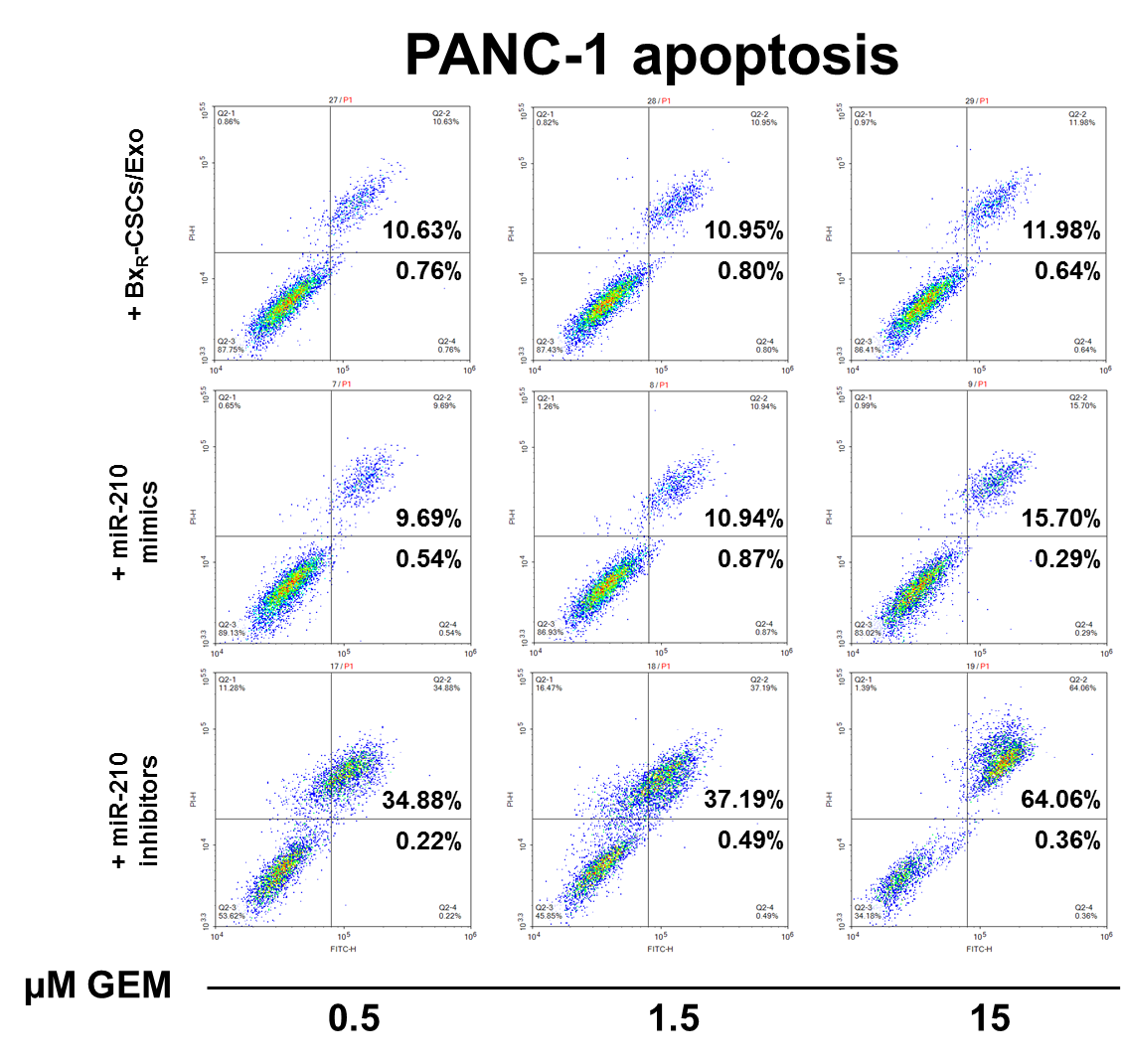

Supplement: Supplementary file 20 — High Resolution Image (TIFF 589 kb) [file 13402_2019_476_MOESM10_ESM.tif]
